# Supplementary figures and images for: Liver ACSM3 deficiency mediates metabolic syndrome via a lauric acid-HNF4α-p38 MAPK axis
Source: EMBO J. 2024 Jan 8;43(4):3. doi: 10.1038/s44318-023-00020-1 (PMC10897460; doi:10.1038/s44318-023-00020-1)

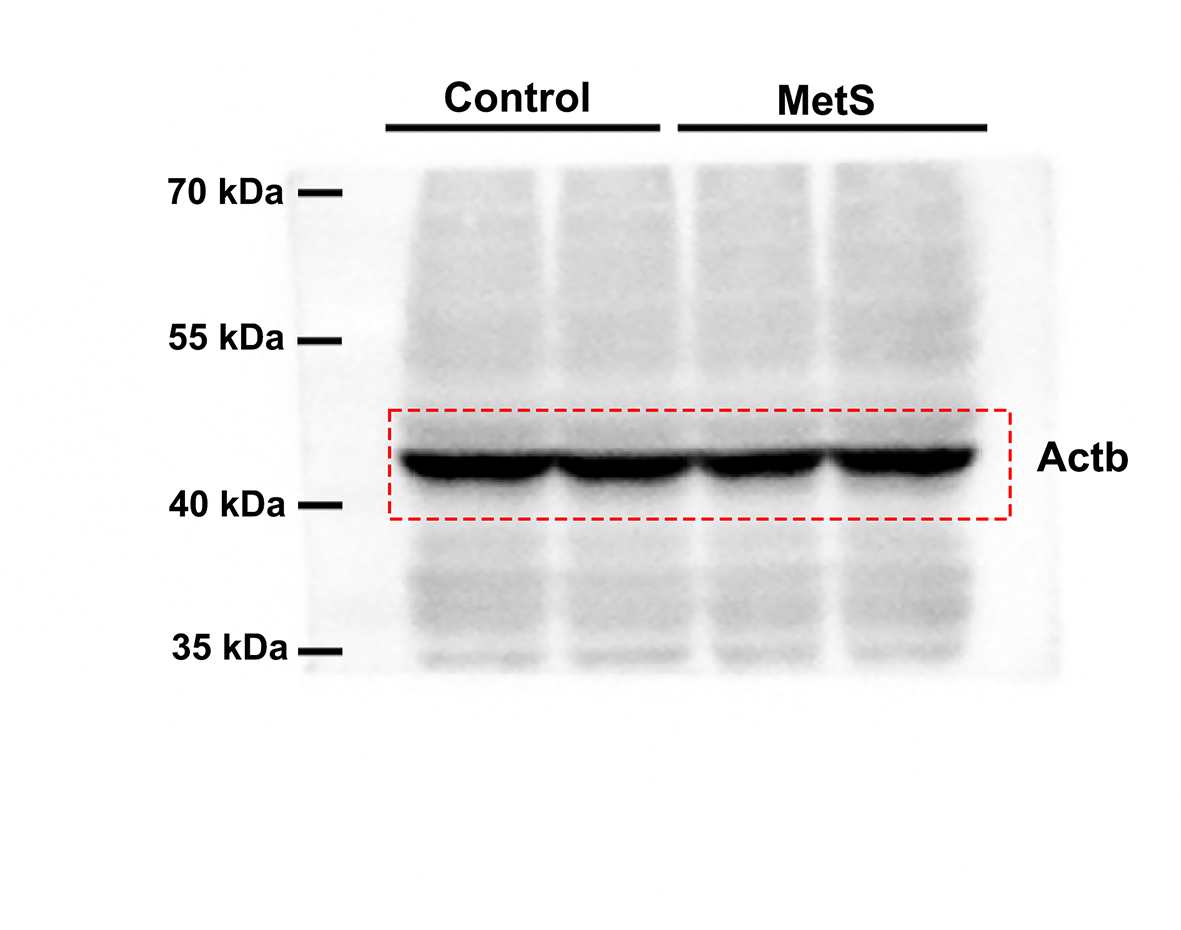

Supplement: Supplementary file 4 — Source Data Fig. 1 [file 44318_2023_20_MOESM4_ESM.zip › Figure 1/1H/Actb.tif]

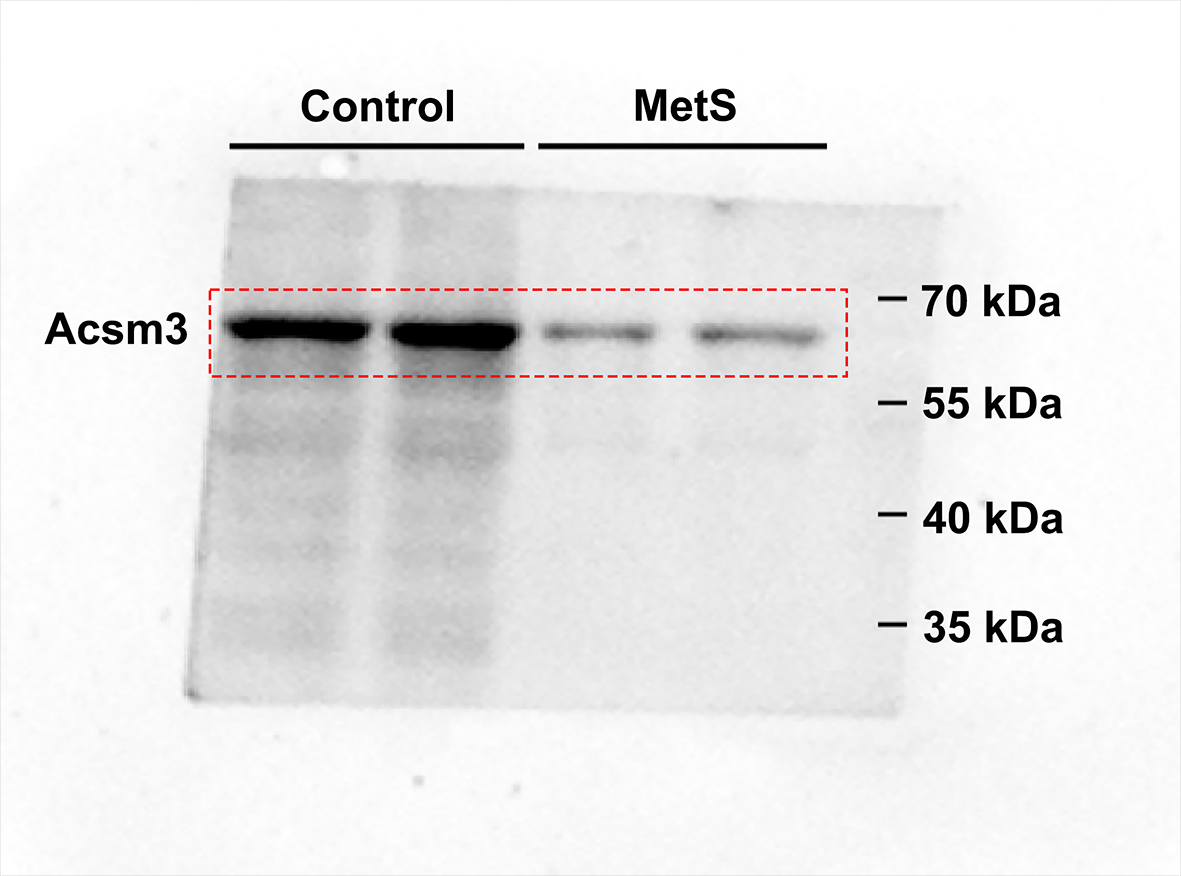

Supplement: Supplementary file 4 — Source Data Fig. 1 [file 44318_2023_20_MOESM4_ESM.zip › Figure 1/1H/Acsm3.tif]

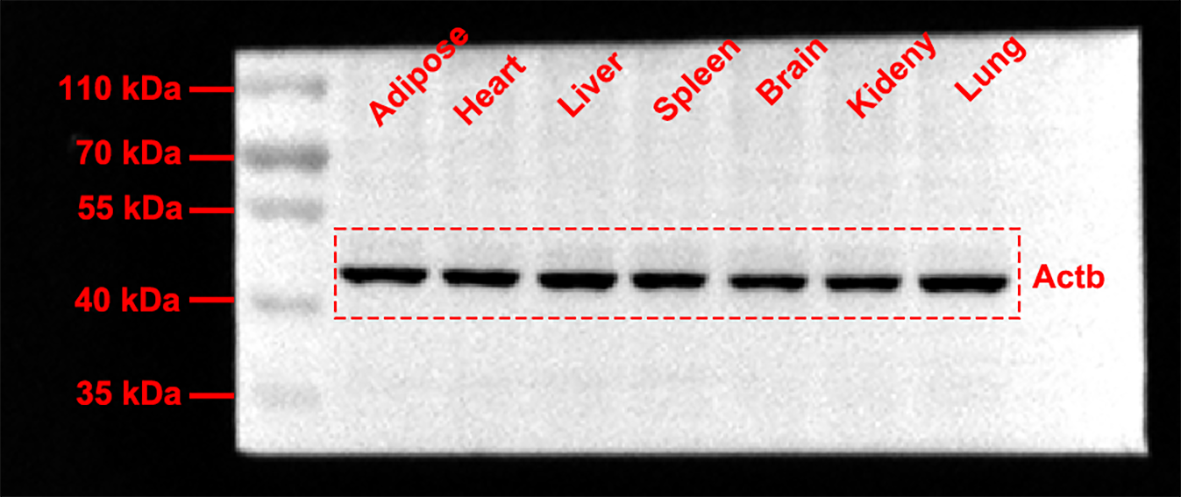

Supplement: Supplementary file 4 — Source Data Fig. 1 [file 44318_2023_20_MOESM4_ESM.zip › Figure 1/1F/Actb.tif]

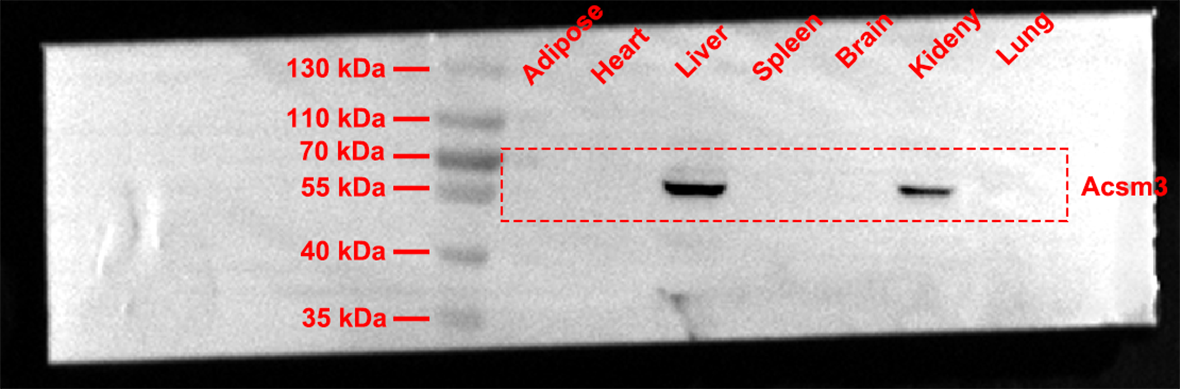

Supplement: Supplementary file 4 — Source Data Fig. 1 [file 44318_2023_20_MOESM4_ESM.zip › Figure 1/1F/Acsm3.tif]

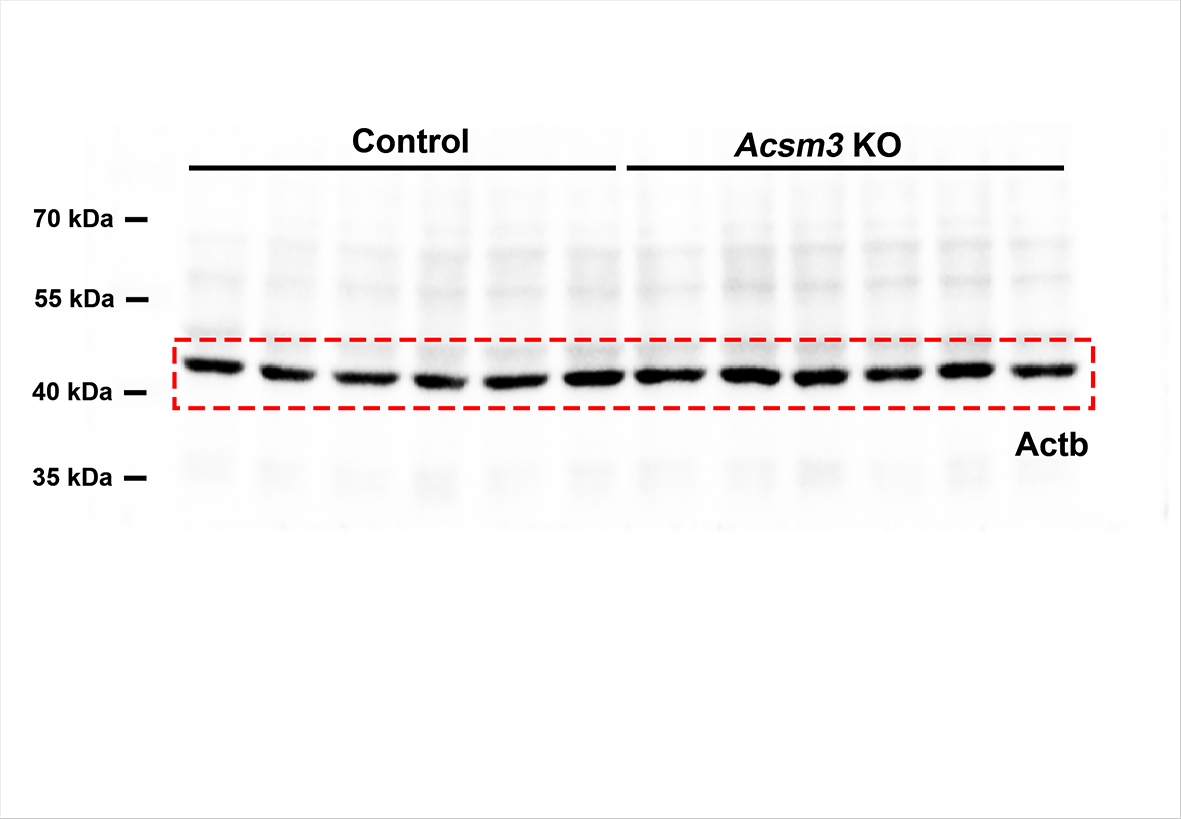

Supplement: Supplementary file 5 — Source Data Fig. 2 [file 44318_2023_20_MOESM5_ESM.zip › Figure 2/2B/Actb.tif]

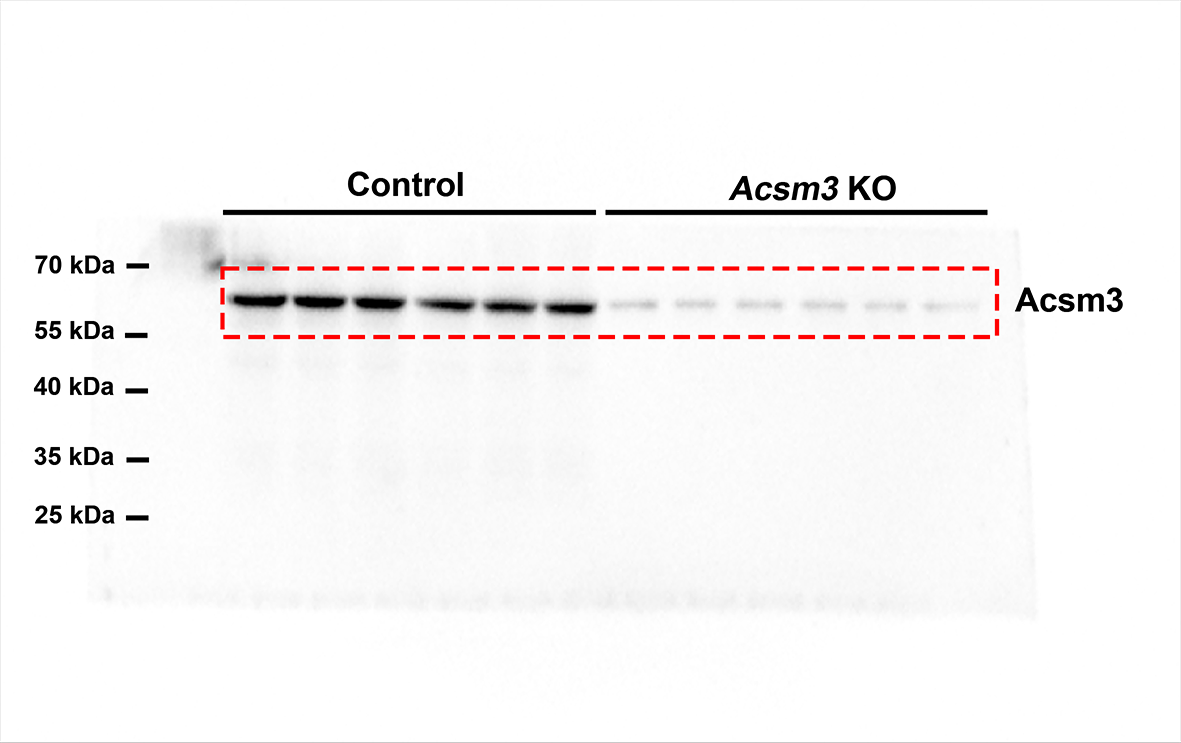

Supplement: Supplementary file 5 — Source Data Fig. 2 [file 44318_2023_20_MOESM5_ESM.zip › Figure 2/2B/Acsm3.tif]

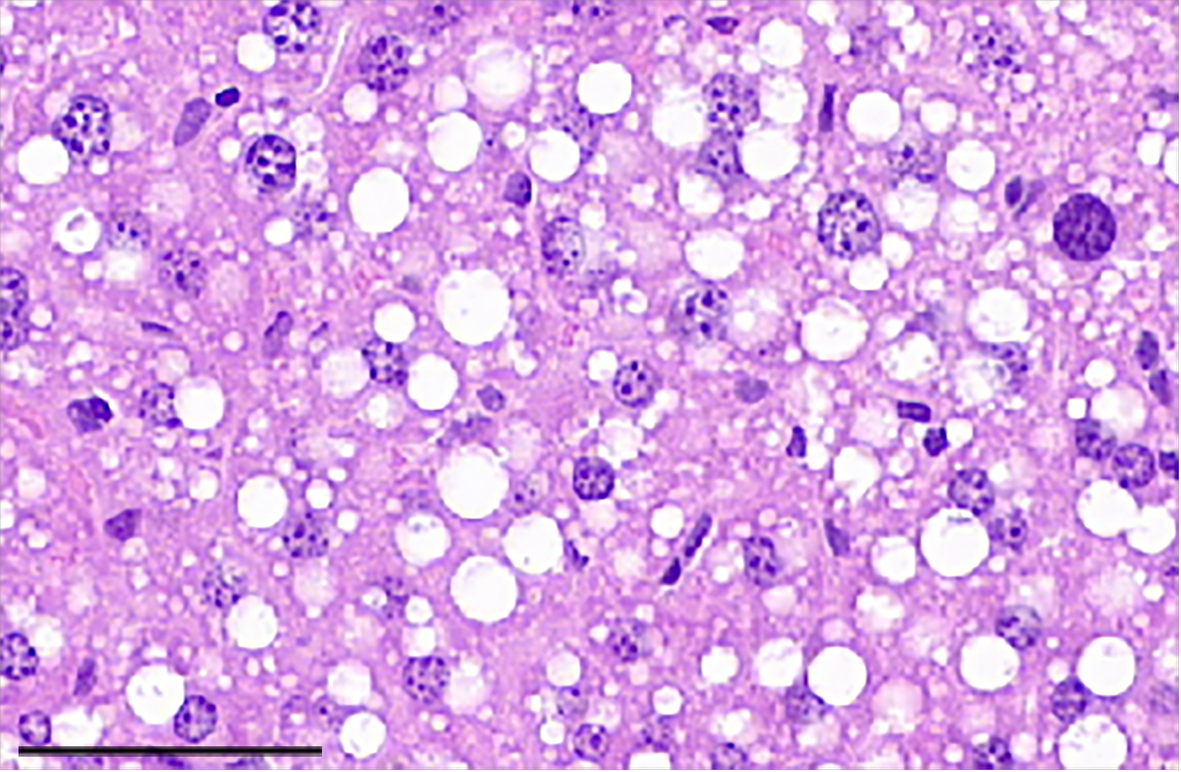

Supplement: Supplementary file 6 — Source Data Fig. 3 [file 44318_2023_20_MOESM6_ESM.zip › Figure 3/3F/HE_Acsm3 KO.tif]

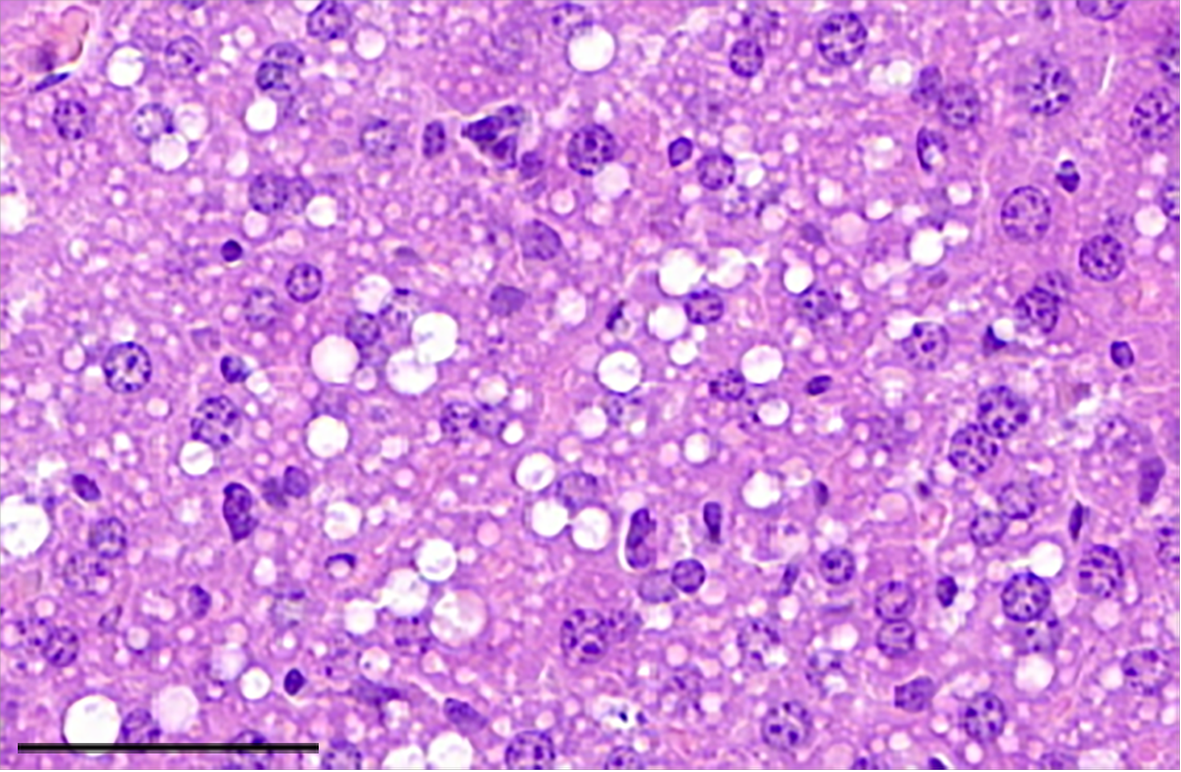

Supplement: Supplementary file 6 — Source Data Fig. 3 [file 44318_2023_20_MOESM6_ESM.zip › Figure 3/3F/HE_Control.tif]

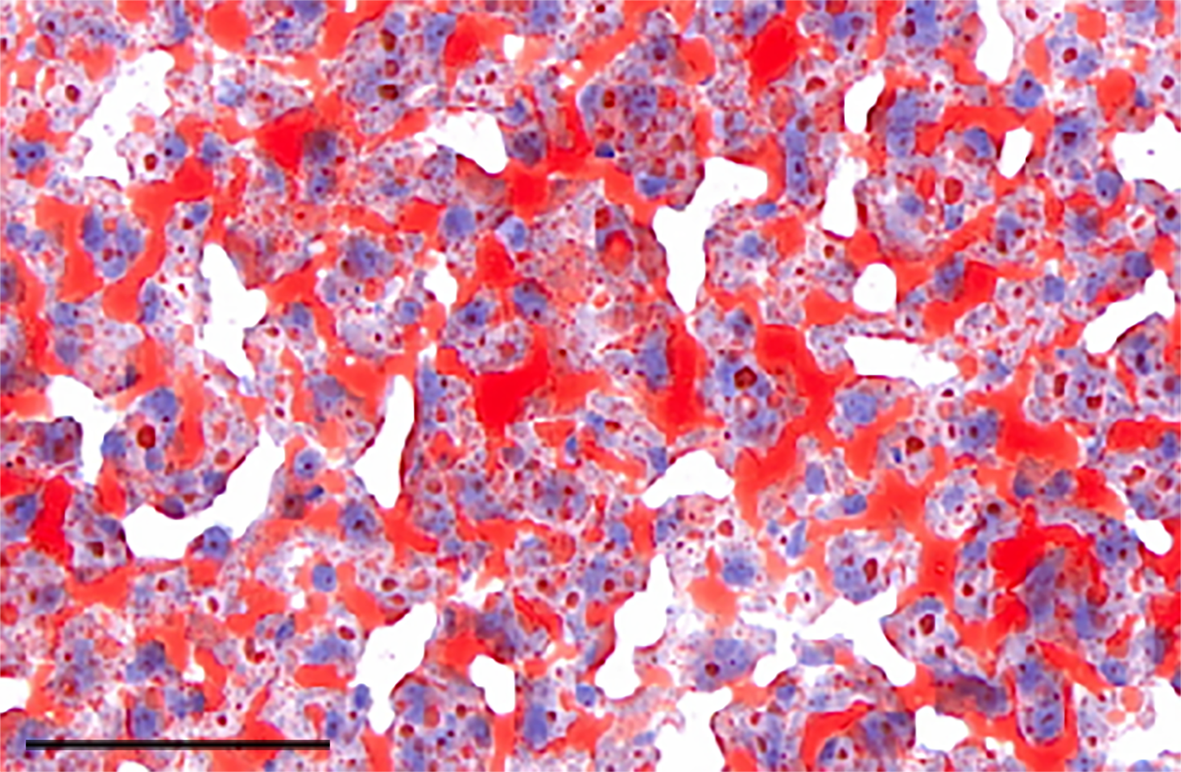

Supplement: Supplementary file 6 — Source Data Fig. 3 [file 44318_2023_20_MOESM6_ESM.zip › Figure 3/3G/Oil red O_Acsm3 KO.tif]

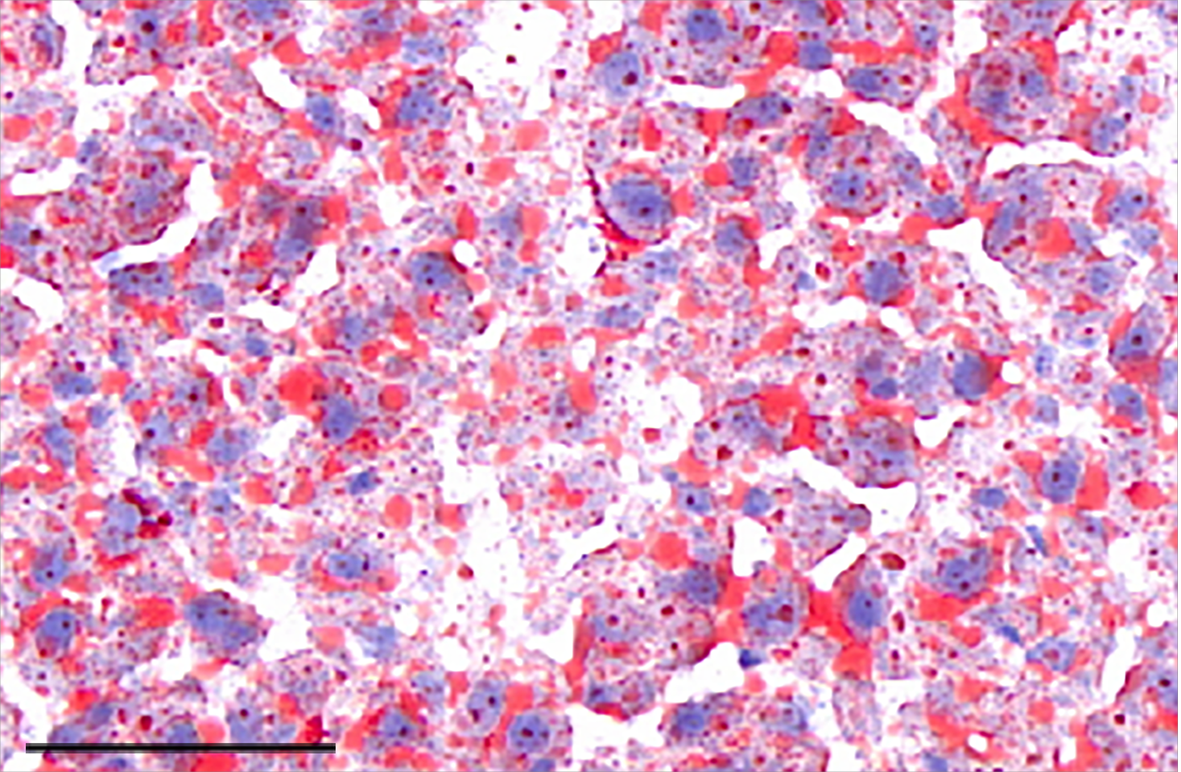

Supplement: Supplementary file 6 — Source Data Fig. 3 [file 44318_2023_20_MOESM6_ESM.zip › Figure 3/3G/Oil red O_Control.tif]

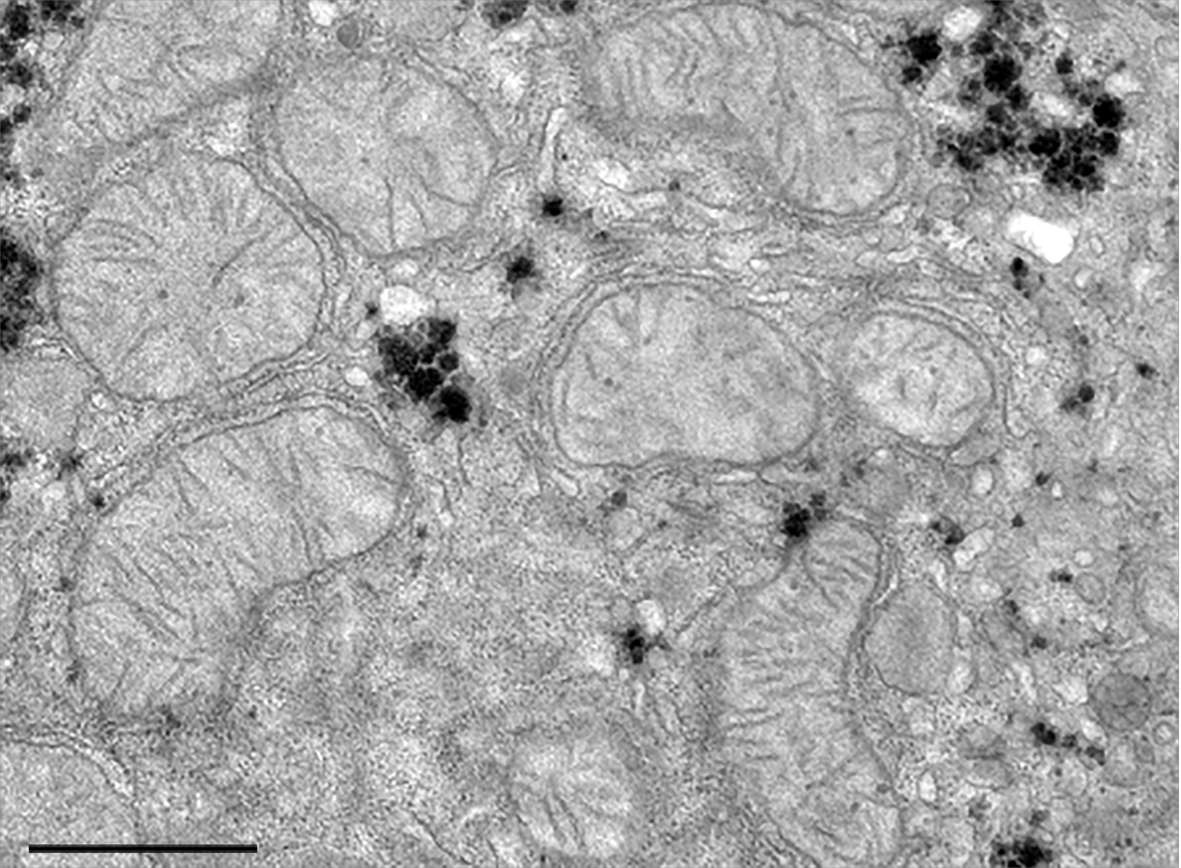

Supplement: Supplementary file 7 — Source Data Fig. 4 [file 44318_2023_20_MOESM7_ESM.zip › Figure 4/4B/Control.tif]

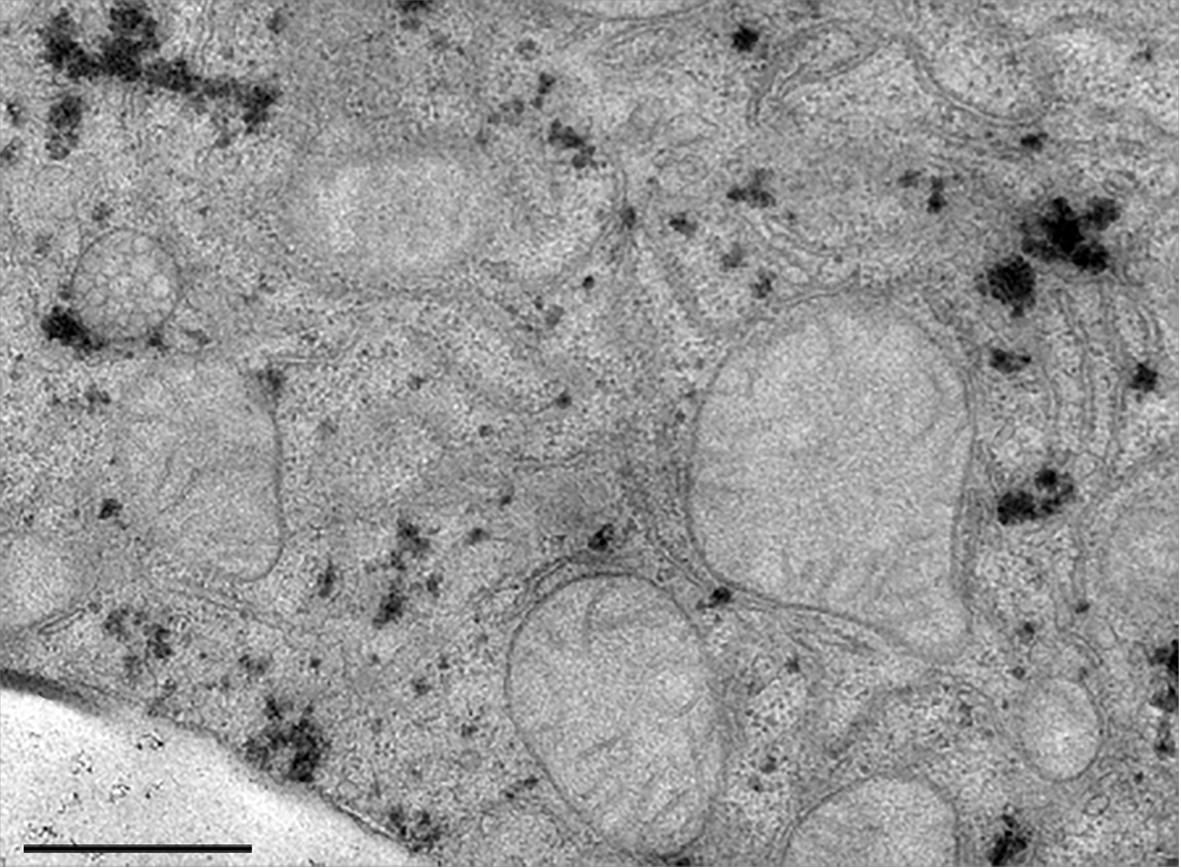

Supplement: Supplementary file 7 — Source Data Fig. 4 [file 44318_2023_20_MOESM7_ESM.zip › Figure 4/4B/Acsm3 KO.tif]

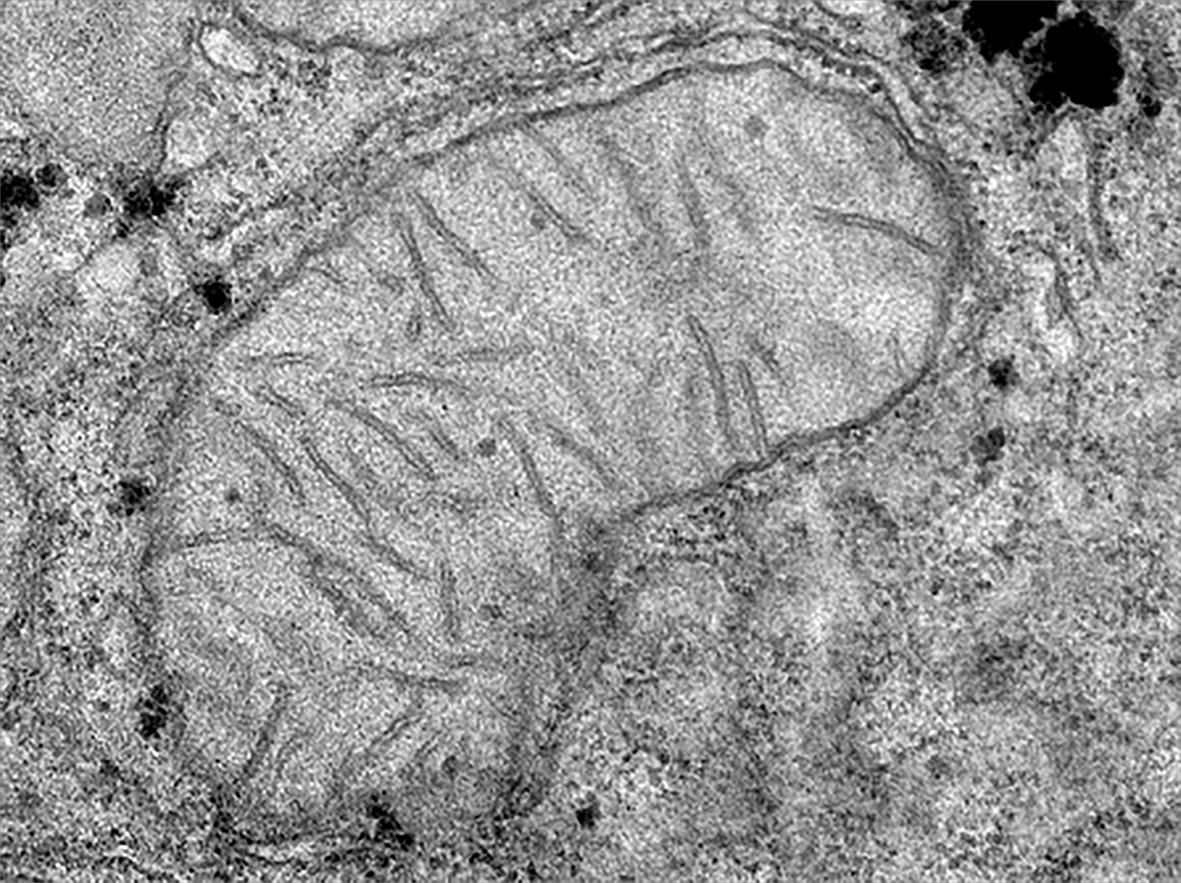

Supplement: Supplementary file 7 — Source Data Fig. 4 [file 44318_2023_20_MOESM7_ESM.zip › Figure 4/4B/Control_enlarge.tif]

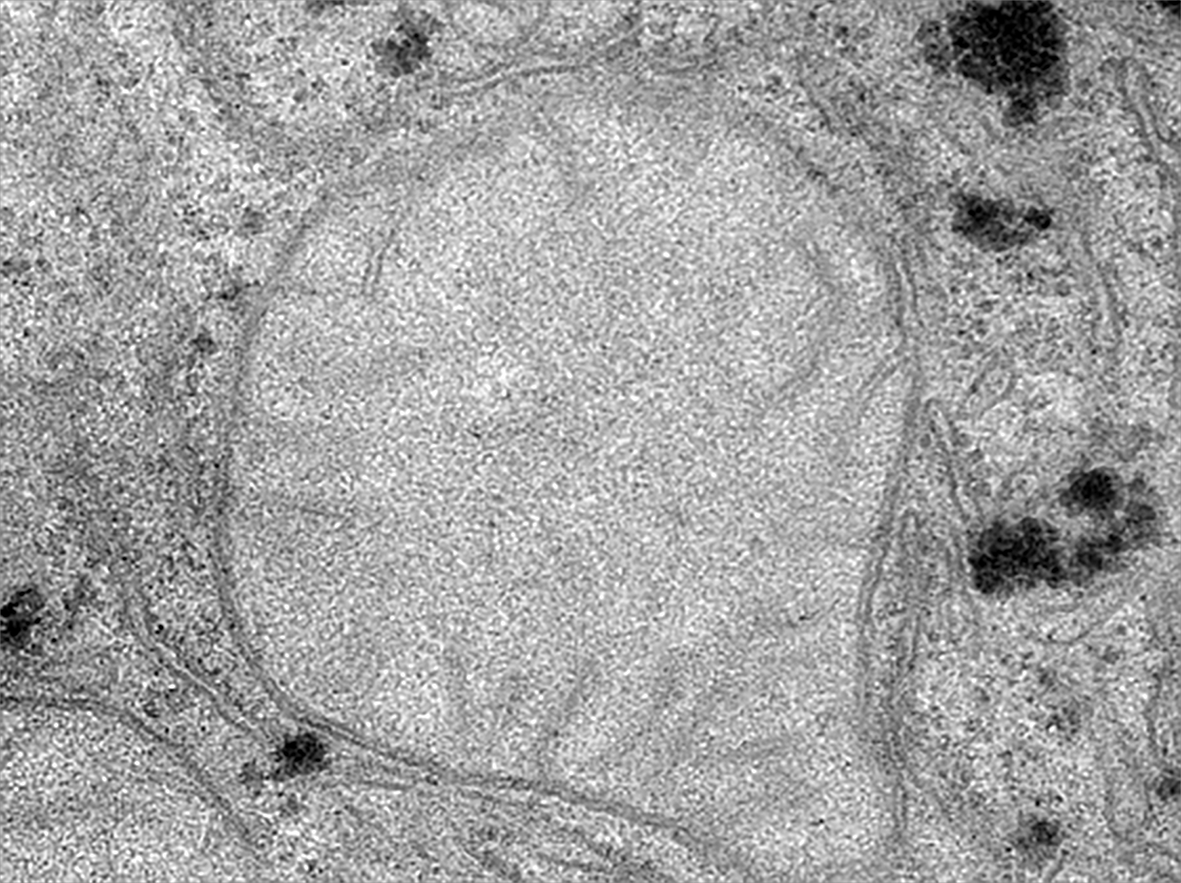

Supplement: Supplementary file 7 — Source Data Fig. 4 [file 44318_2023_20_MOESM7_ESM.zip › Figure 4/4B/Acsm3 KO_enlarge.tif]

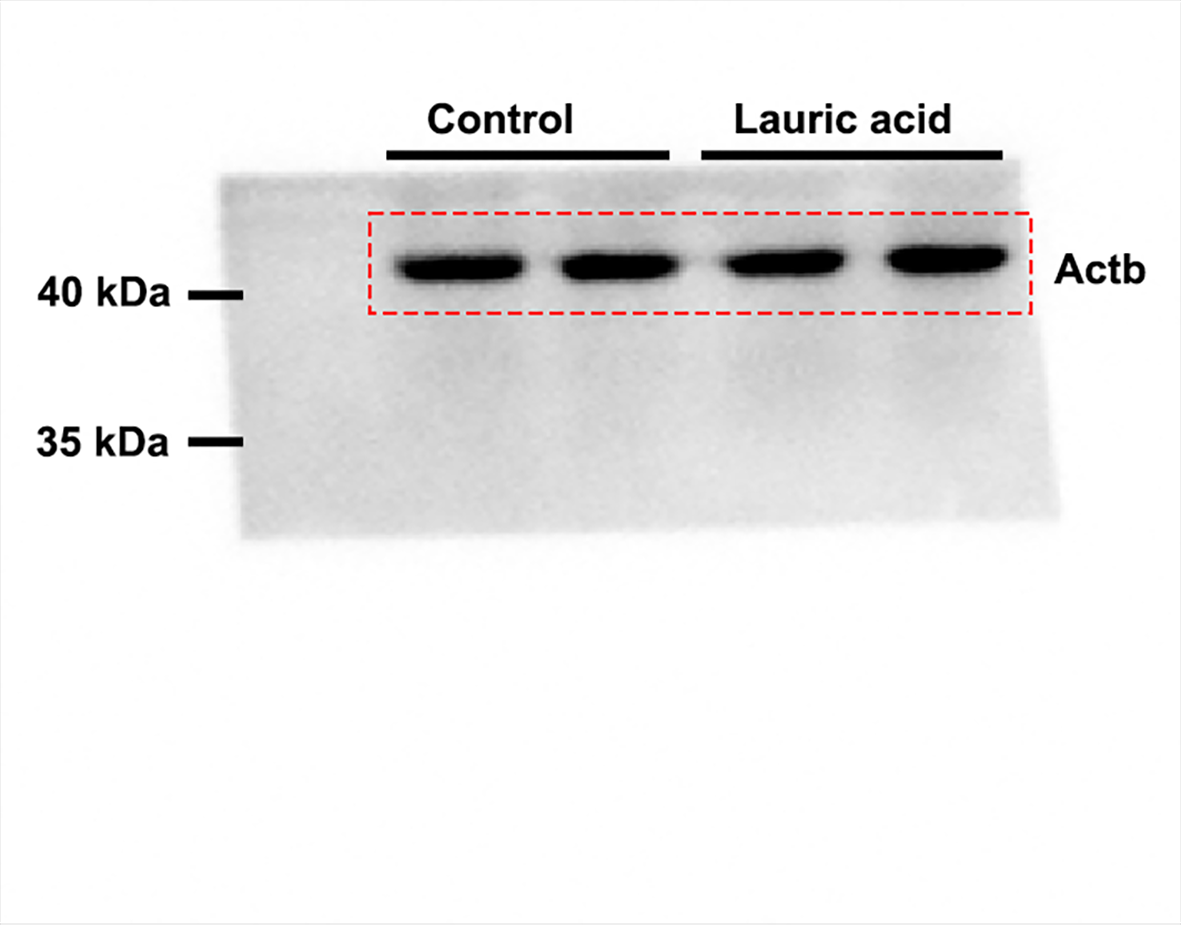

Supplement: Supplementary file 8 — Source Data Fig. 5 [file 44318_2023_20_MOESM8_ESM.zip › Figure 5/5I/Actb.tif]

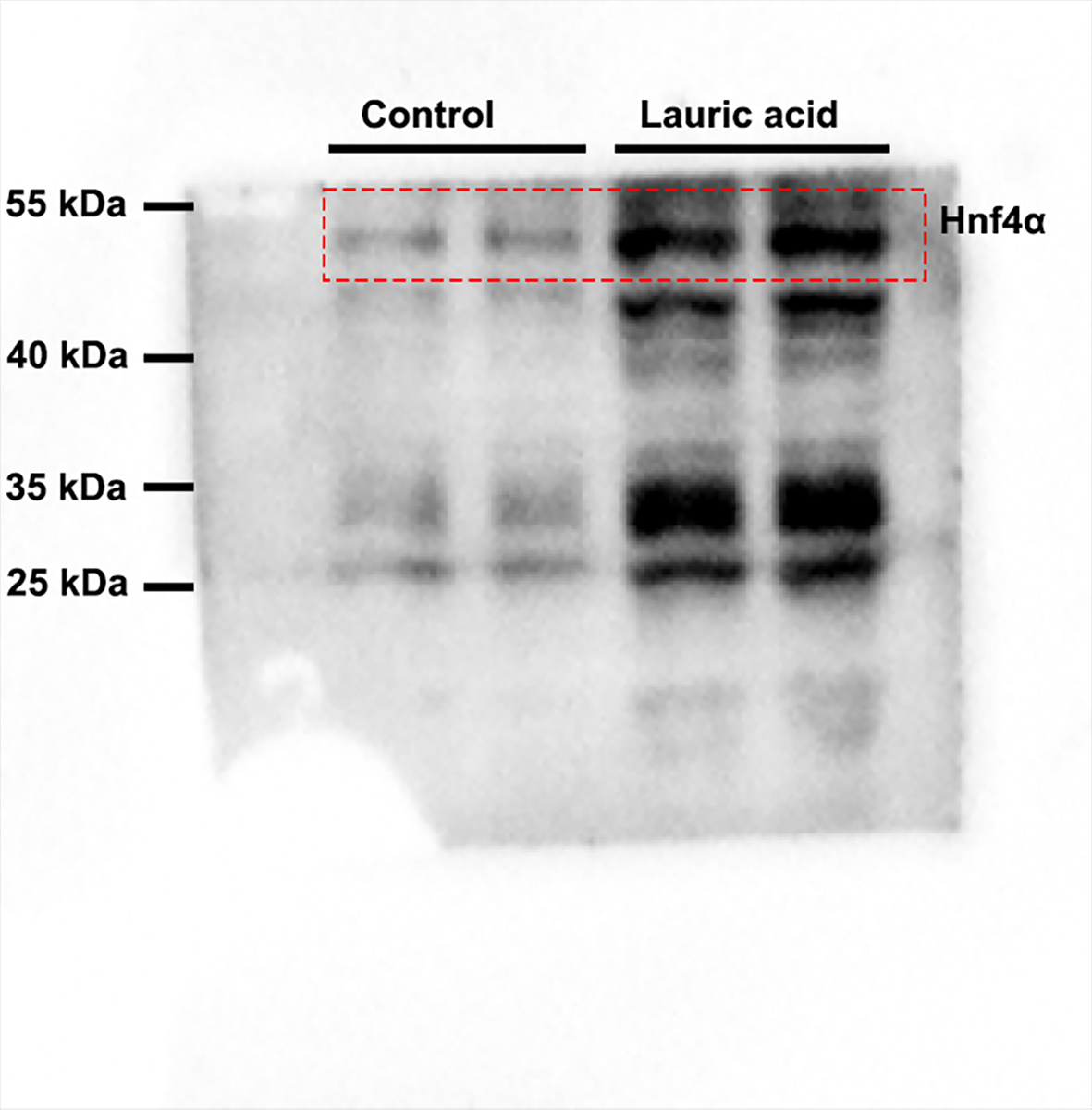

Supplement: Supplementary file 8 — Source Data Fig. 5 [file 44318_2023_20_MOESM8_ESM.zip › Figure 5/5I/Hnf4╬▒.tif]

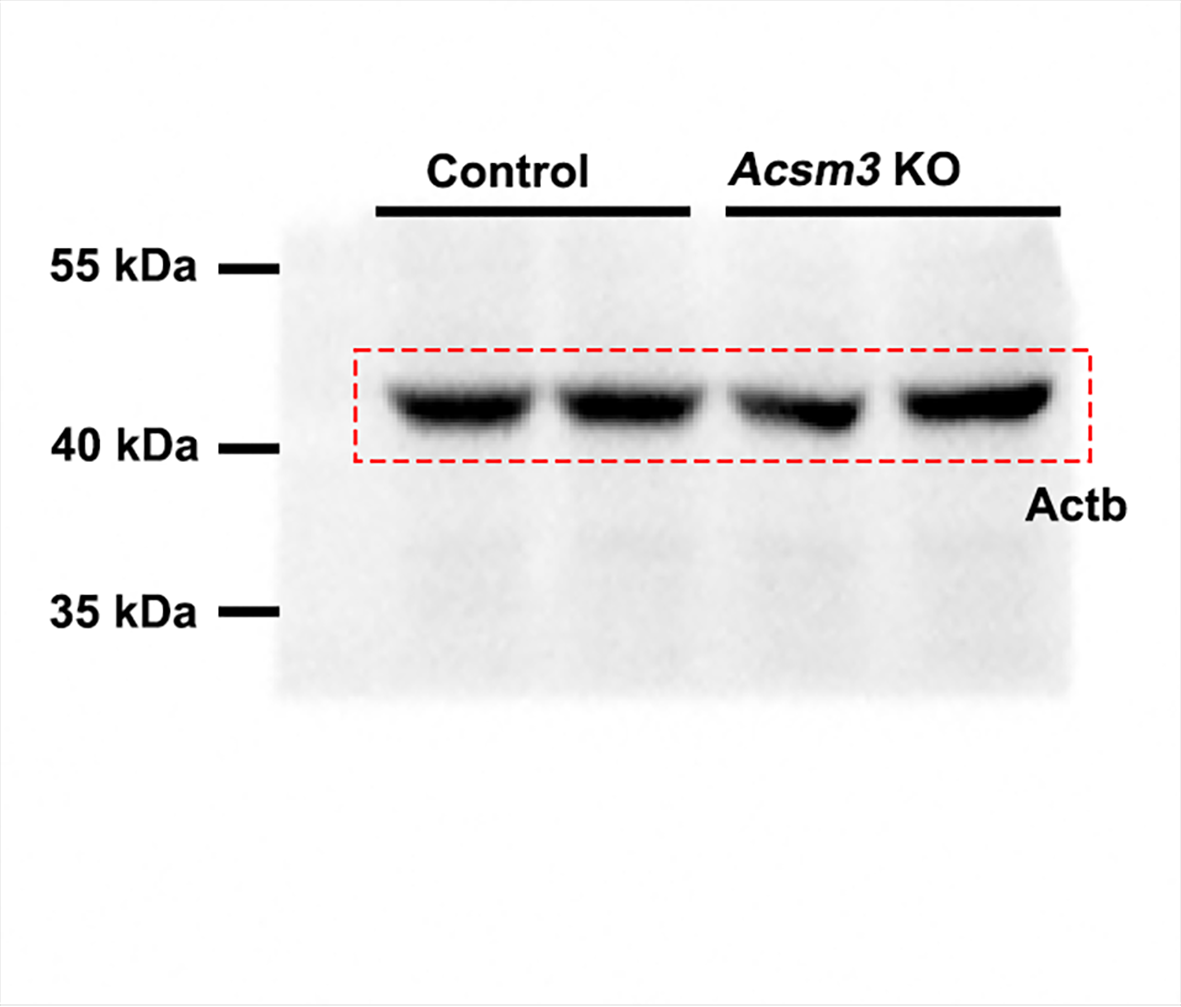

Supplement: Supplementary file 8 — Source Data Fig. 5 [file 44318_2023_20_MOESM8_ESM.zip › Figure 5/5H/Actb.tif]

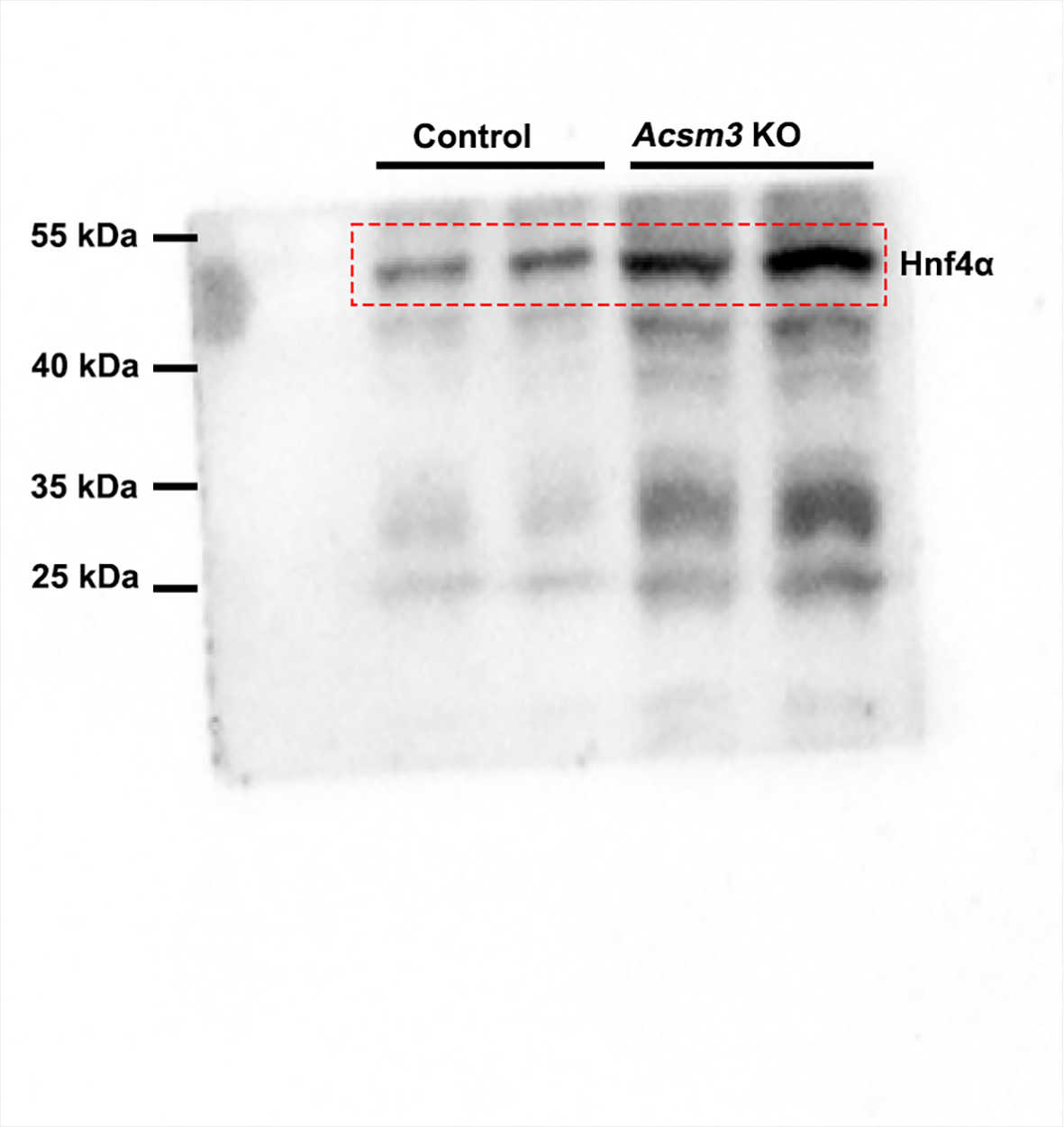

Supplement: Supplementary file 8 — Source Data Fig. 5 [file 44318_2023_20_MOESM8_ESM.zip › Figure 5/5H/Hnf4╬▒.tif]

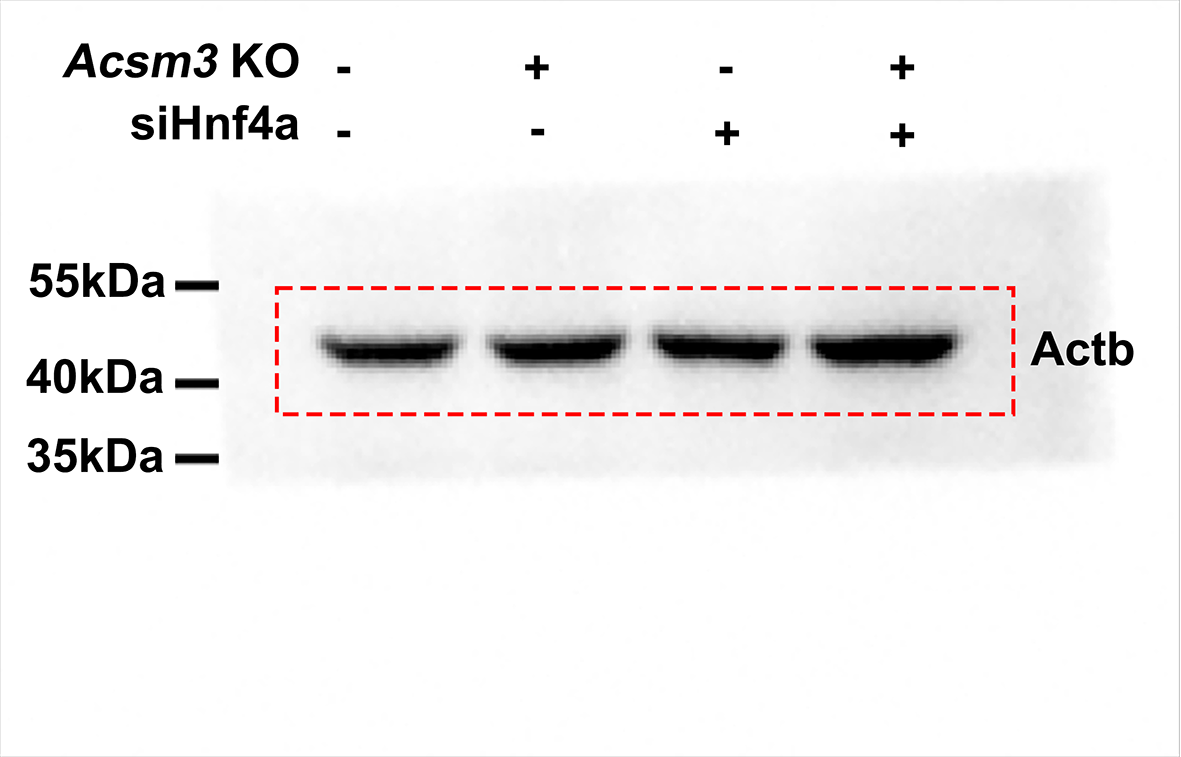

Supplement: Supplementary file 8 — Source Data Fig. 5 [file 44318_2023_20_MOESM8_ESM.zip › Figure 5/5M/Actb.tif]

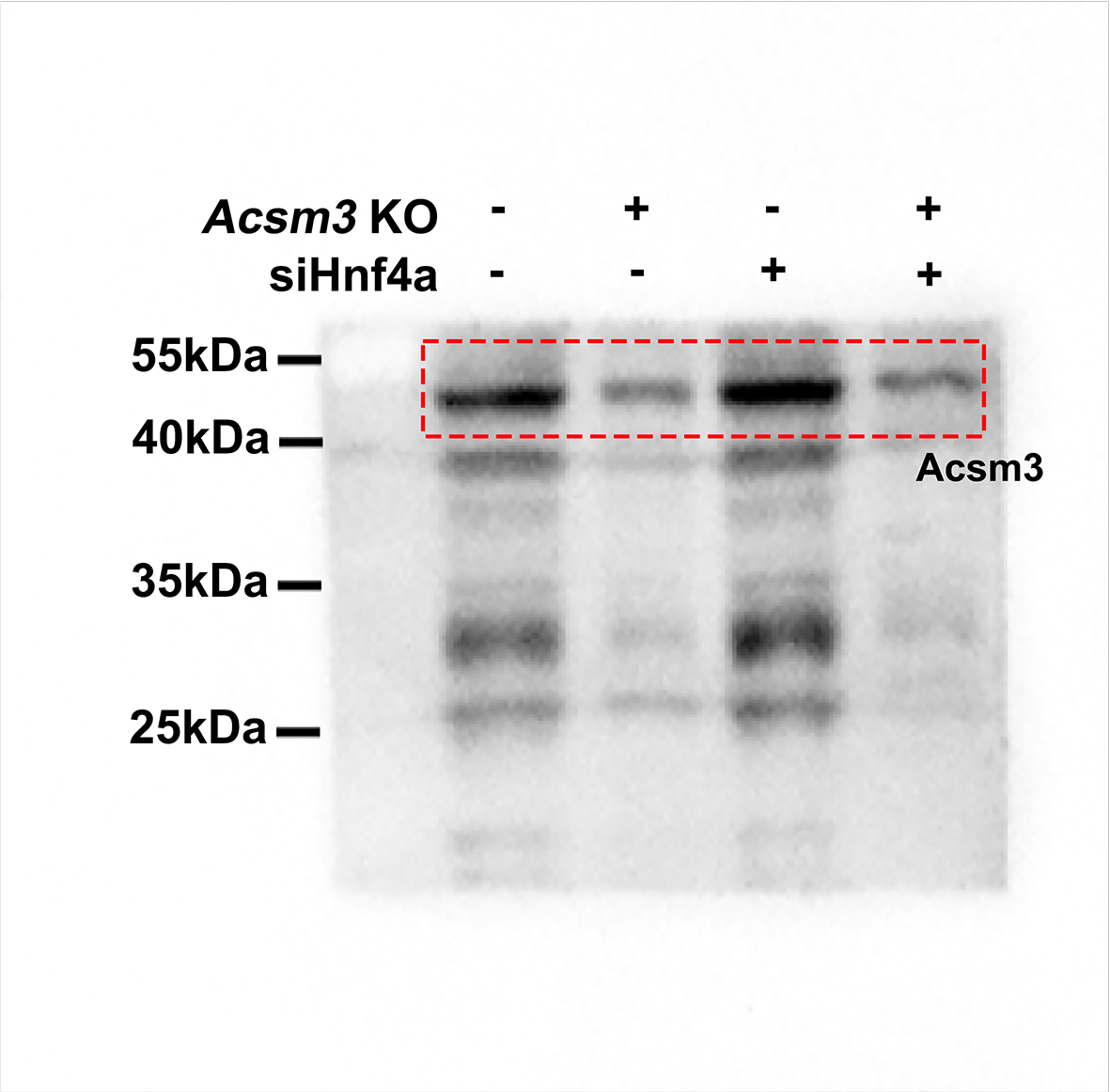

Supplement: Supplementary file 8 — Source Data Fig. 5 [file 44318_2023_20_MOESM8_ESM.zip › Figure 5/5M/Acsm3.tif]

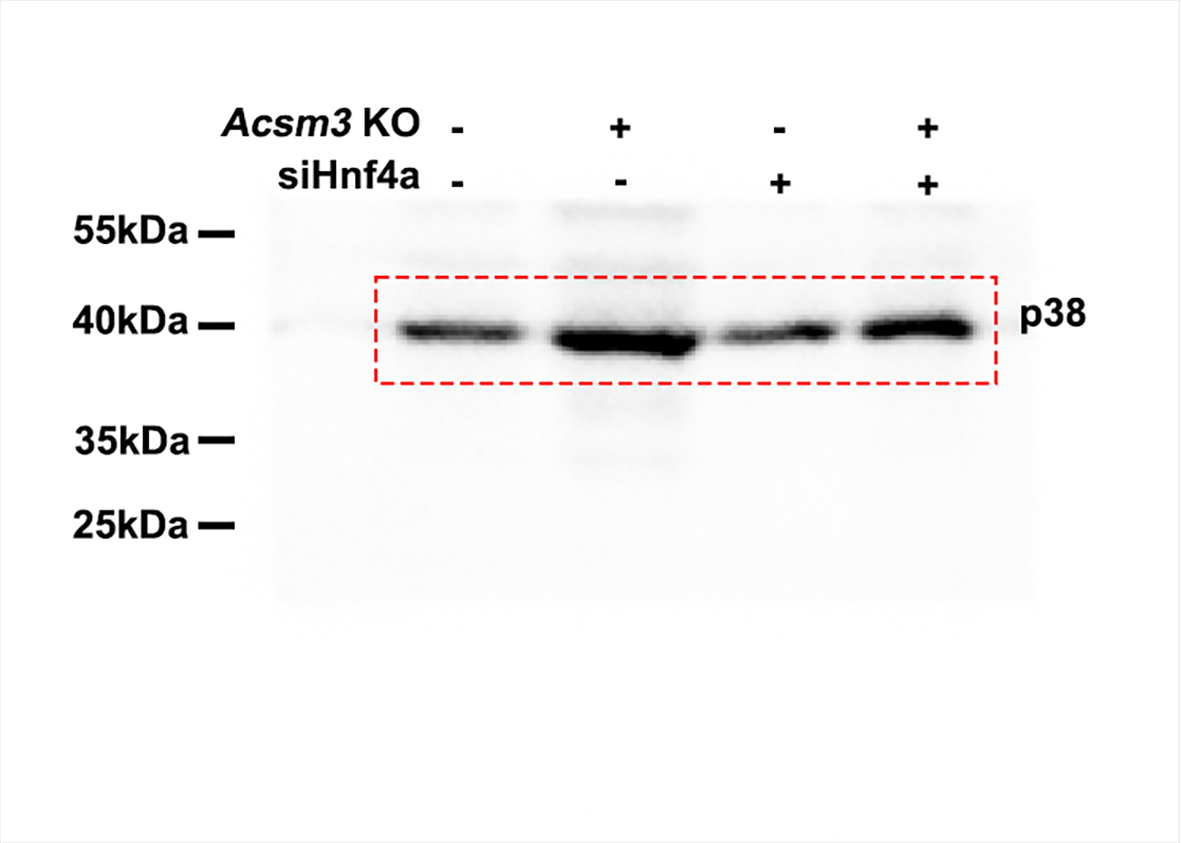

Supplement: Supplementary file 8 — Source Data Fig. 5 [file 44318_2023_20_MOESM8_ESM.zip › Figure 5/5M/p38.tif]

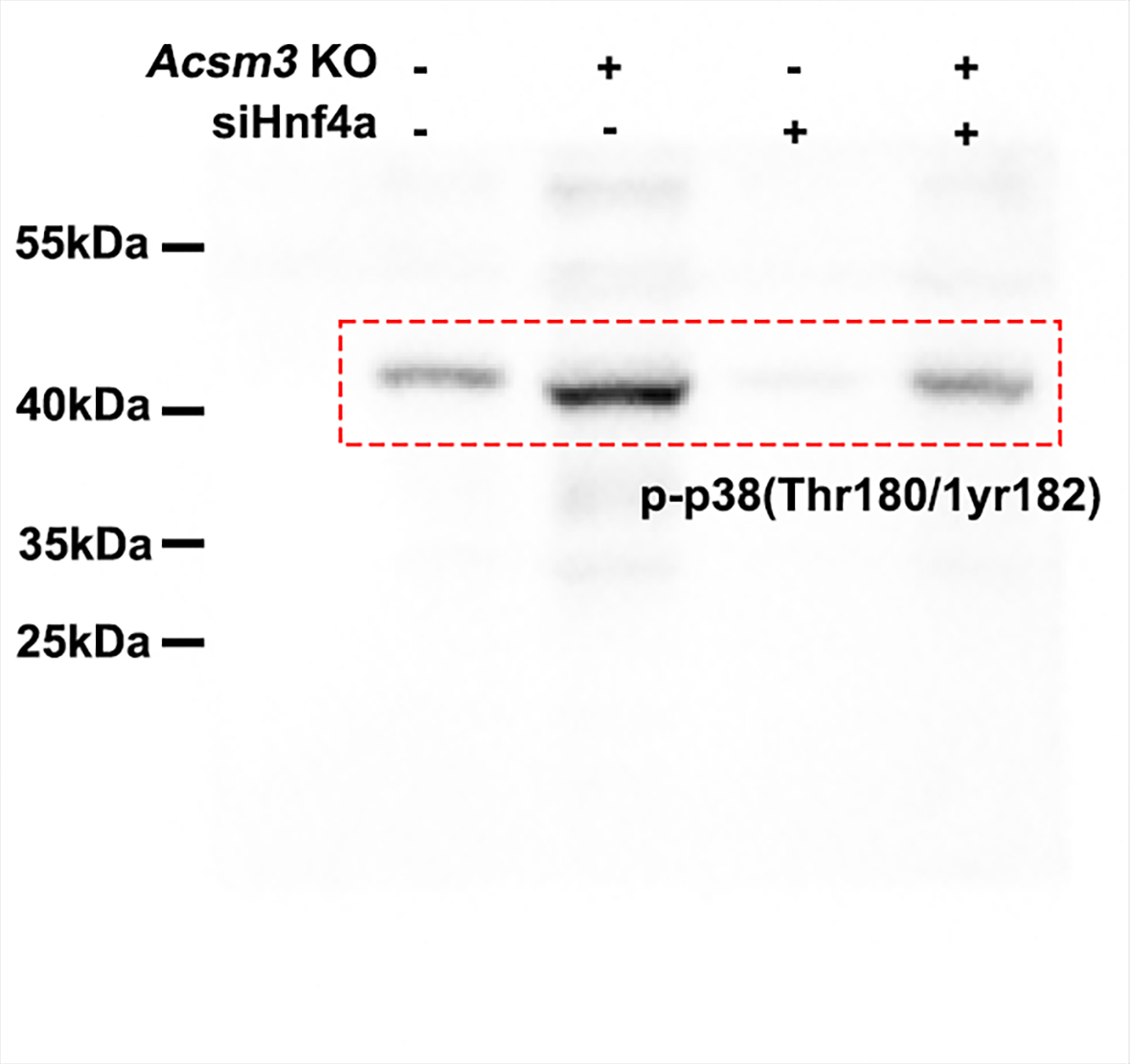

Supplement: Supplementary file 8 — Source Data Fig. 5 [file 44318_2023_20_MOESM8_ESM.zip › Figure 5/5M/p-p38(Thr180 Tyr182).tif]

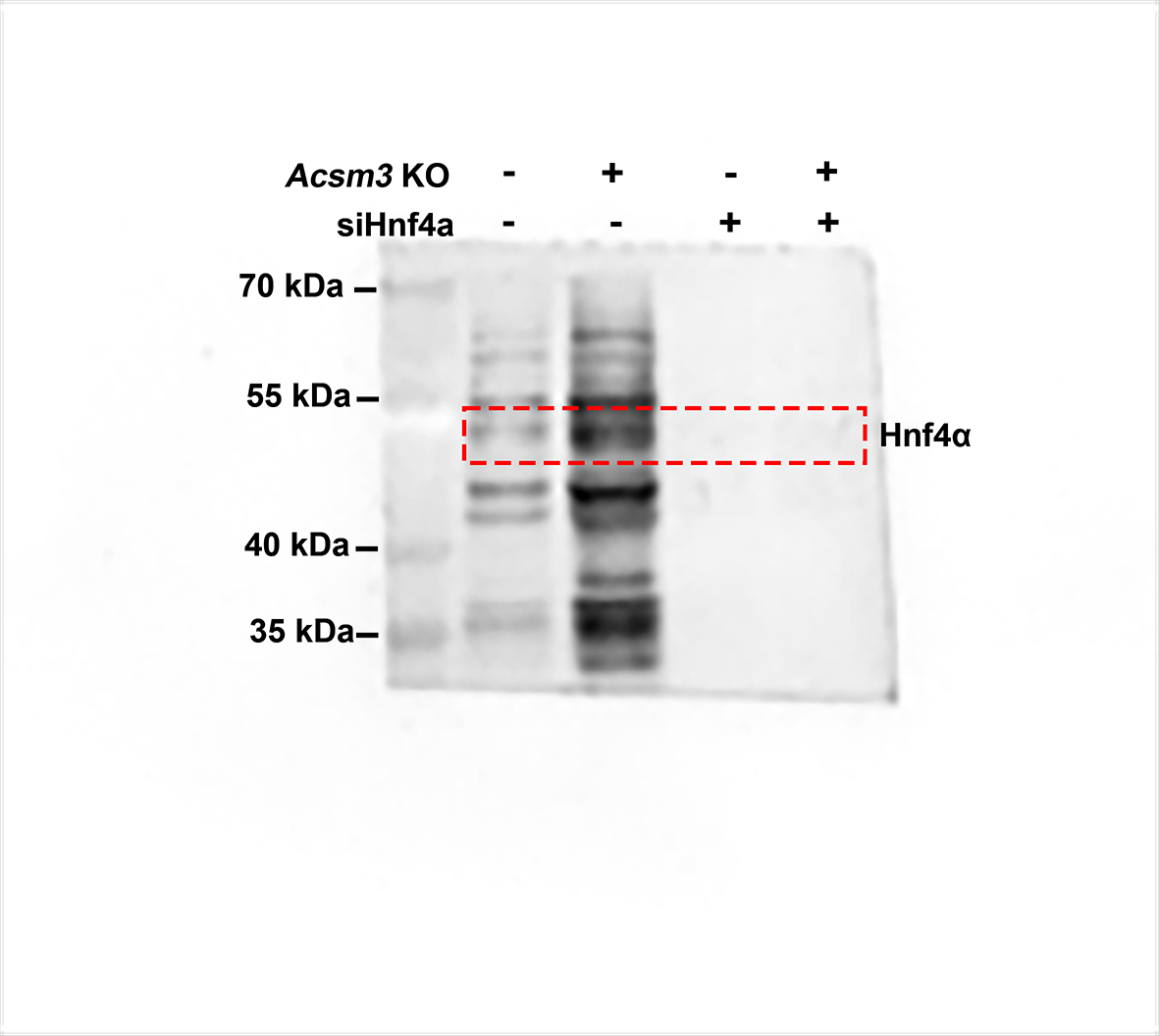

Supplement: Supplementary file 8 — Source Data Fig. 5 [file 44318_2023_20_MOESM8_ESM.zip › Figure 5/5M/Hnf4╬▒.tif]

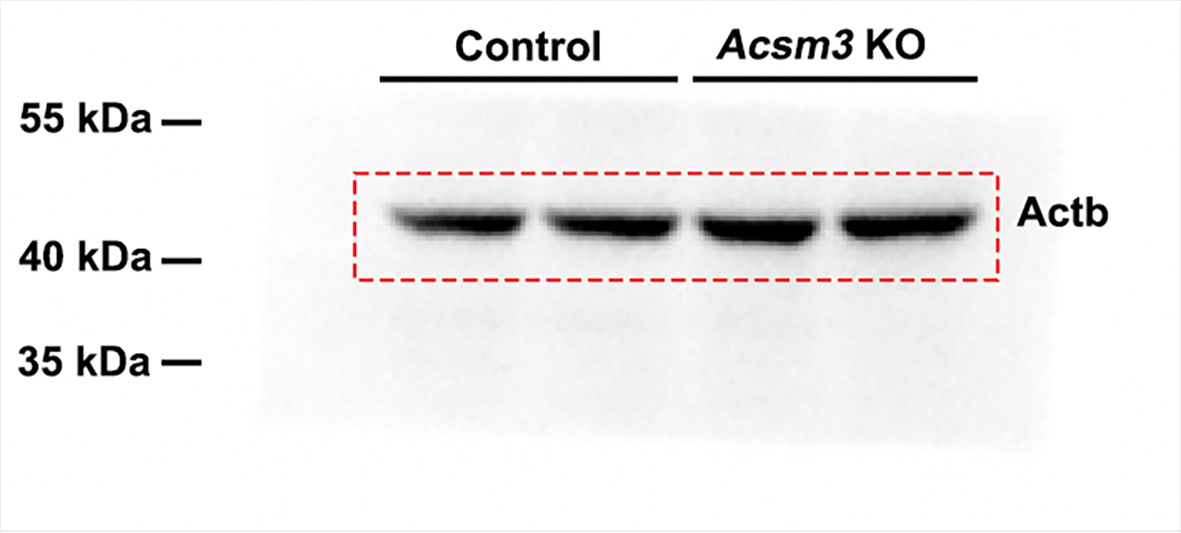

Supplement: Supplementary file 8 — Source Data Fig. 5 [file 44318_2023_20_MOESM8_ESM.zip › Figure 5/5C/Actb.tif]

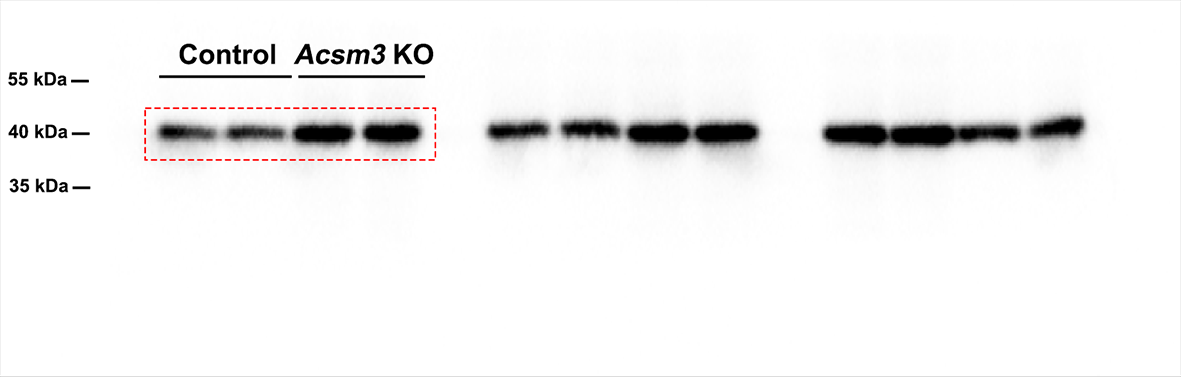

Supplement: Supplementary file 8 — Source Data Fig. 5 [file 44318_2023_20_MOESM8_ESM.zip › Figure 5/5C/p38.tif]

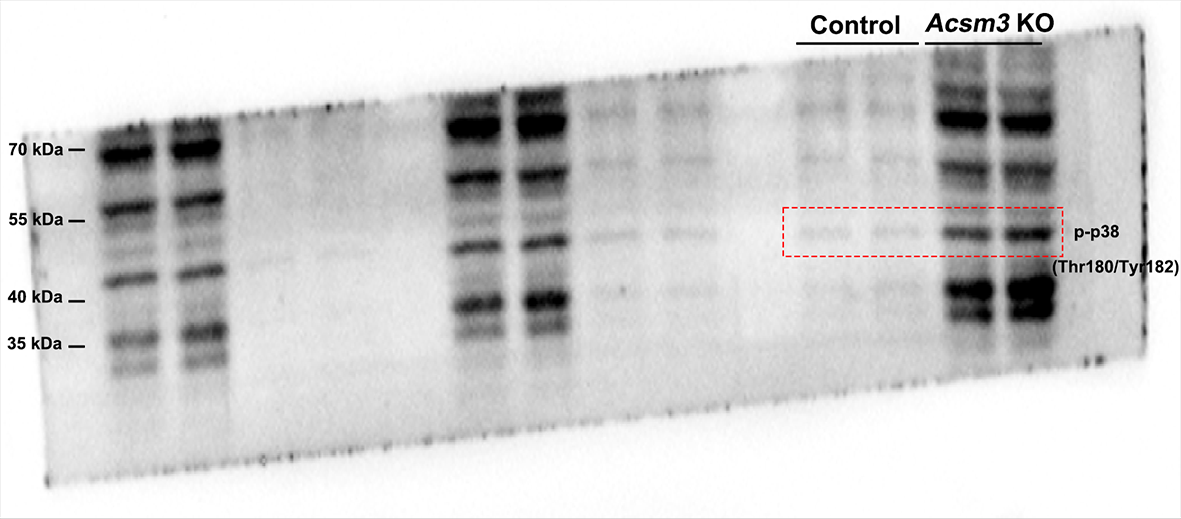

Supplement: Supplementary file 8 — Source Data Fig. 5 [file 44318_2023_20_MOESM8_ESM.zip › Figure 5/5C/p-p38(Thr180 Tyr182).tif]

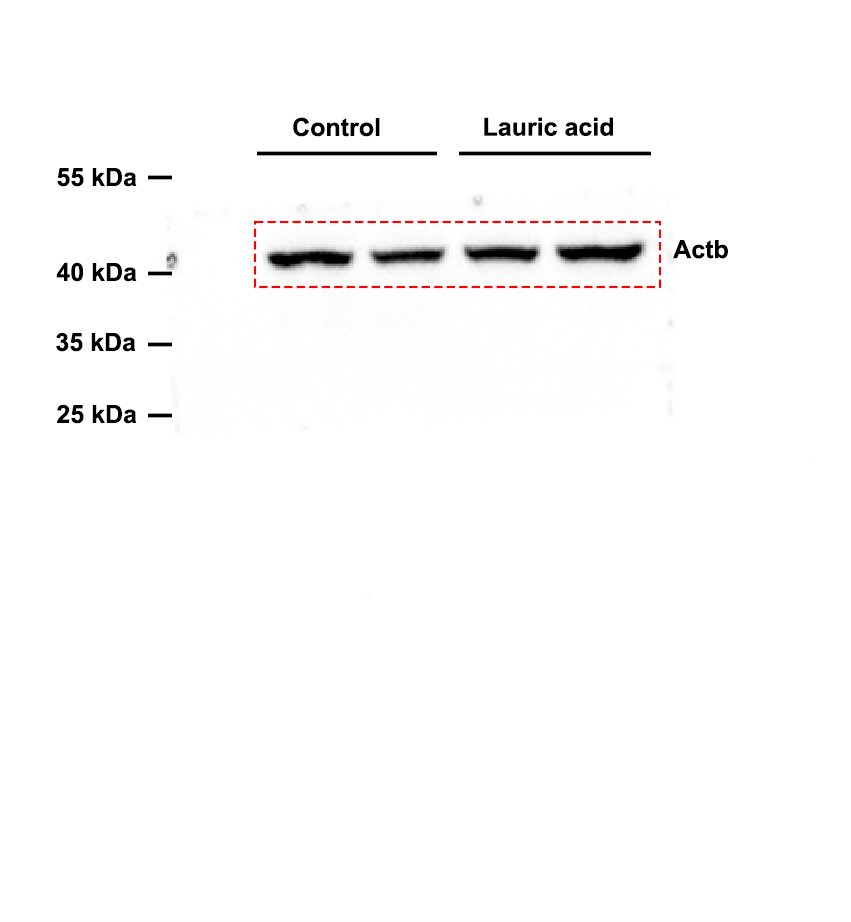

Supplement: Supplementary file 8 — Source Data Fig. 5 [file 44318_2023_20_MOESM8_ESM.zip › Figure 5/5D/Actb.tif]

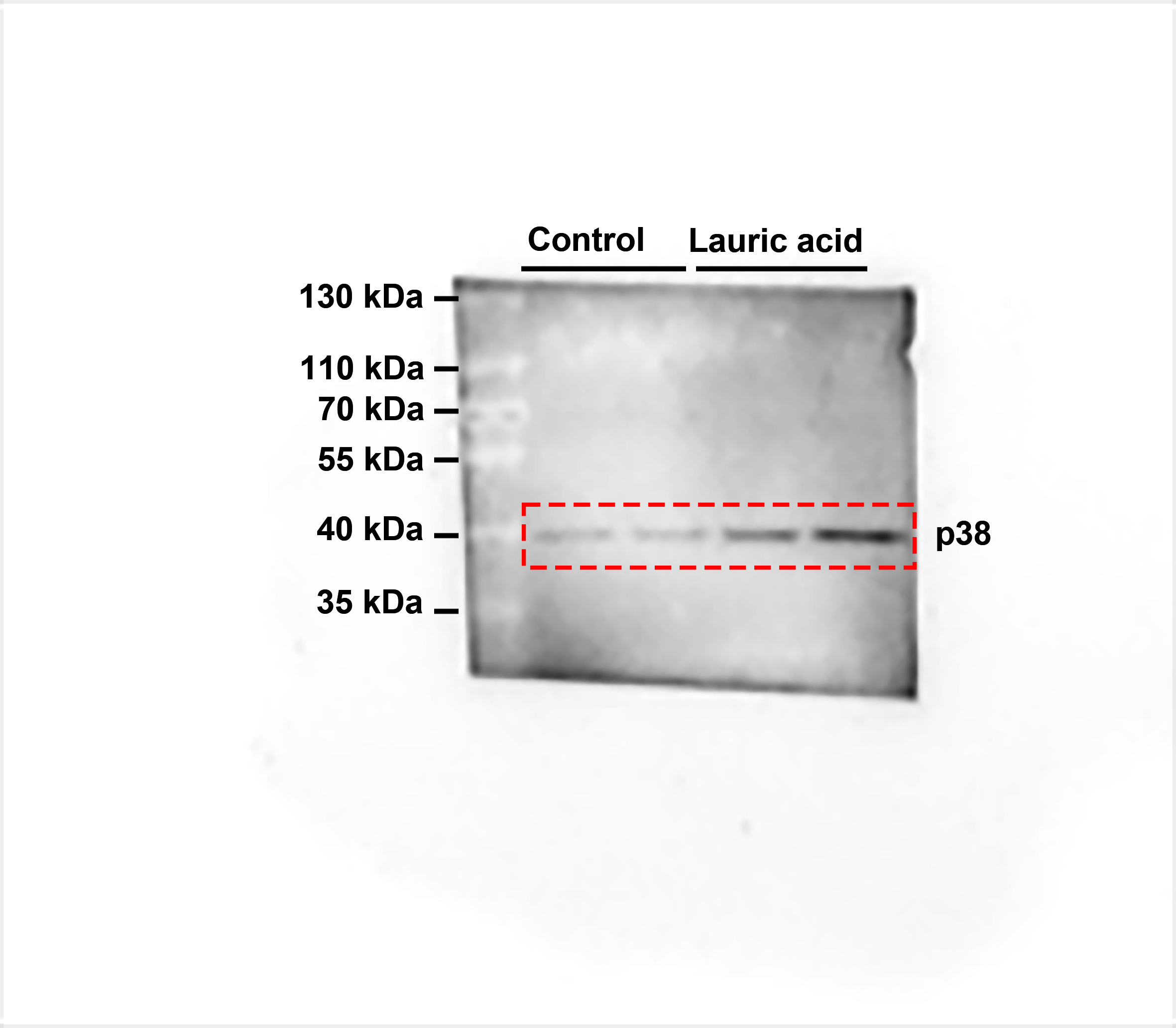

Supplement: Supplementary file 8 — Source Data Fig. 5 [file 44318_2023_20_MOESM8_ESM.zip › Figure 5/5D/p38.tif]

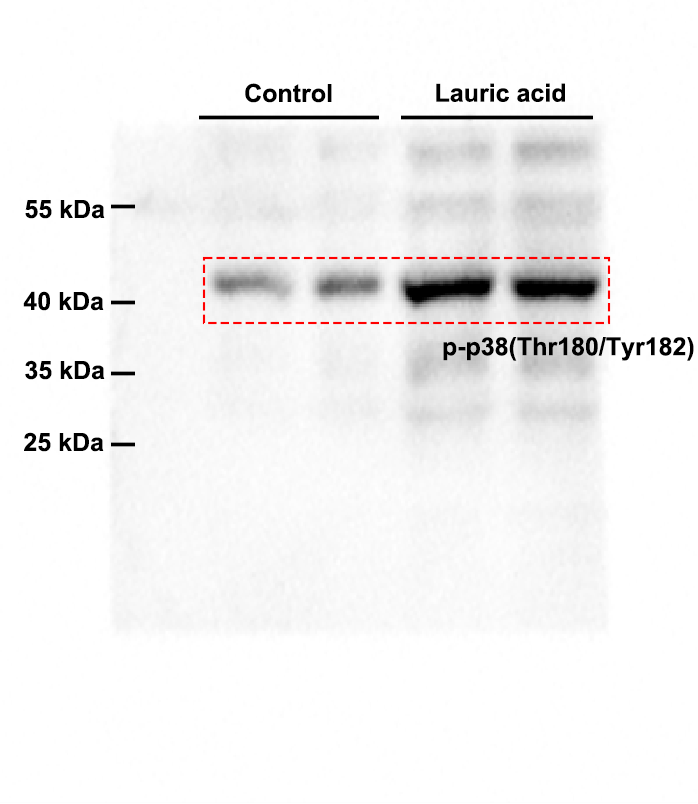

Supplement: Supplementary file 8 — Source Data Fig. 5 [file 44318_2023_20_MOESM8_ESM.zip › Figure 5/5D/p-p38(Thr180 Tyr182).tif]

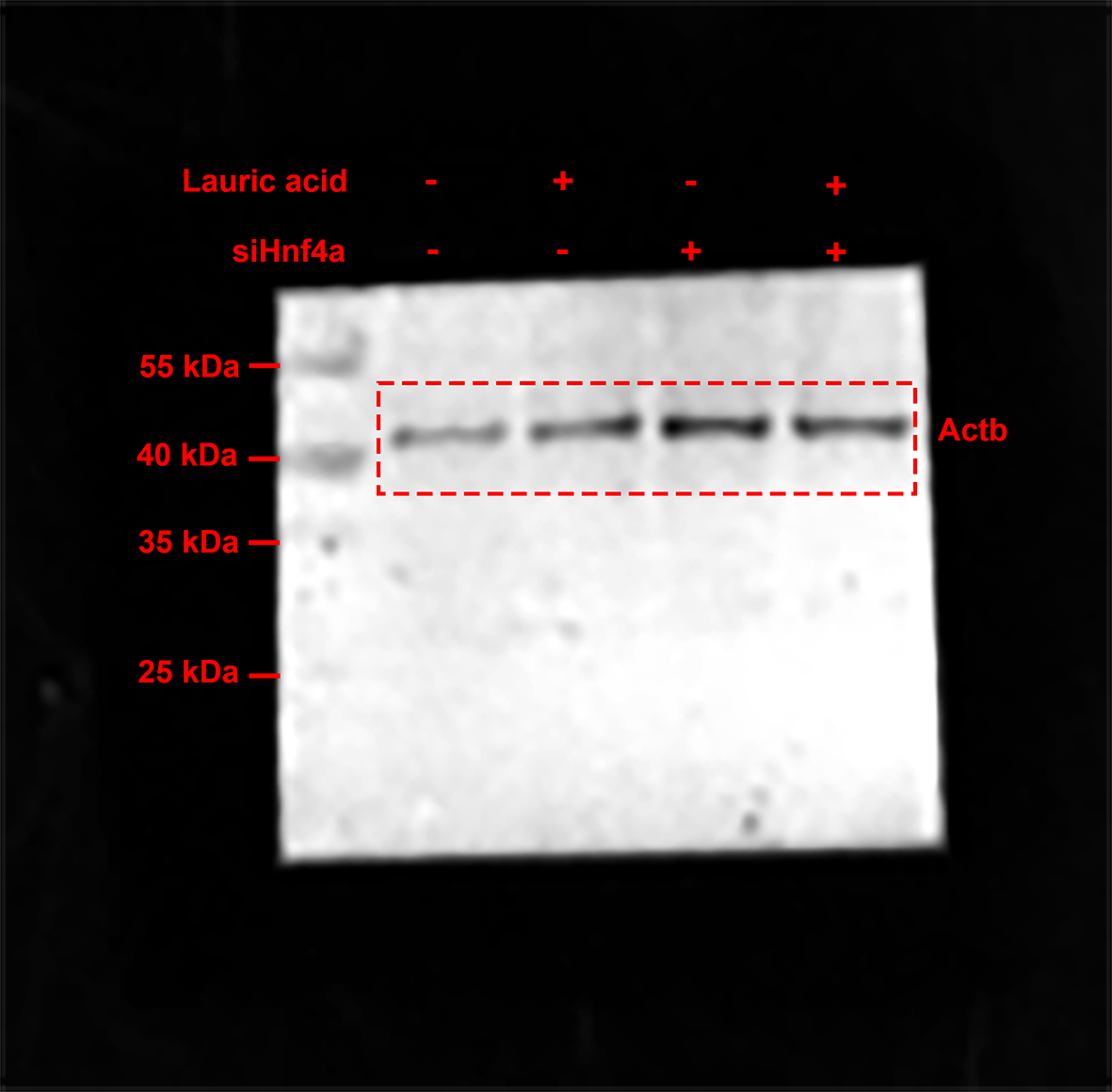

Supplement: Supplementary file 8 — Source Data Fig. 5 [file 44318_2023_20_MOESM8_ESM.zip › Figure 5/5L/Actb.tif]

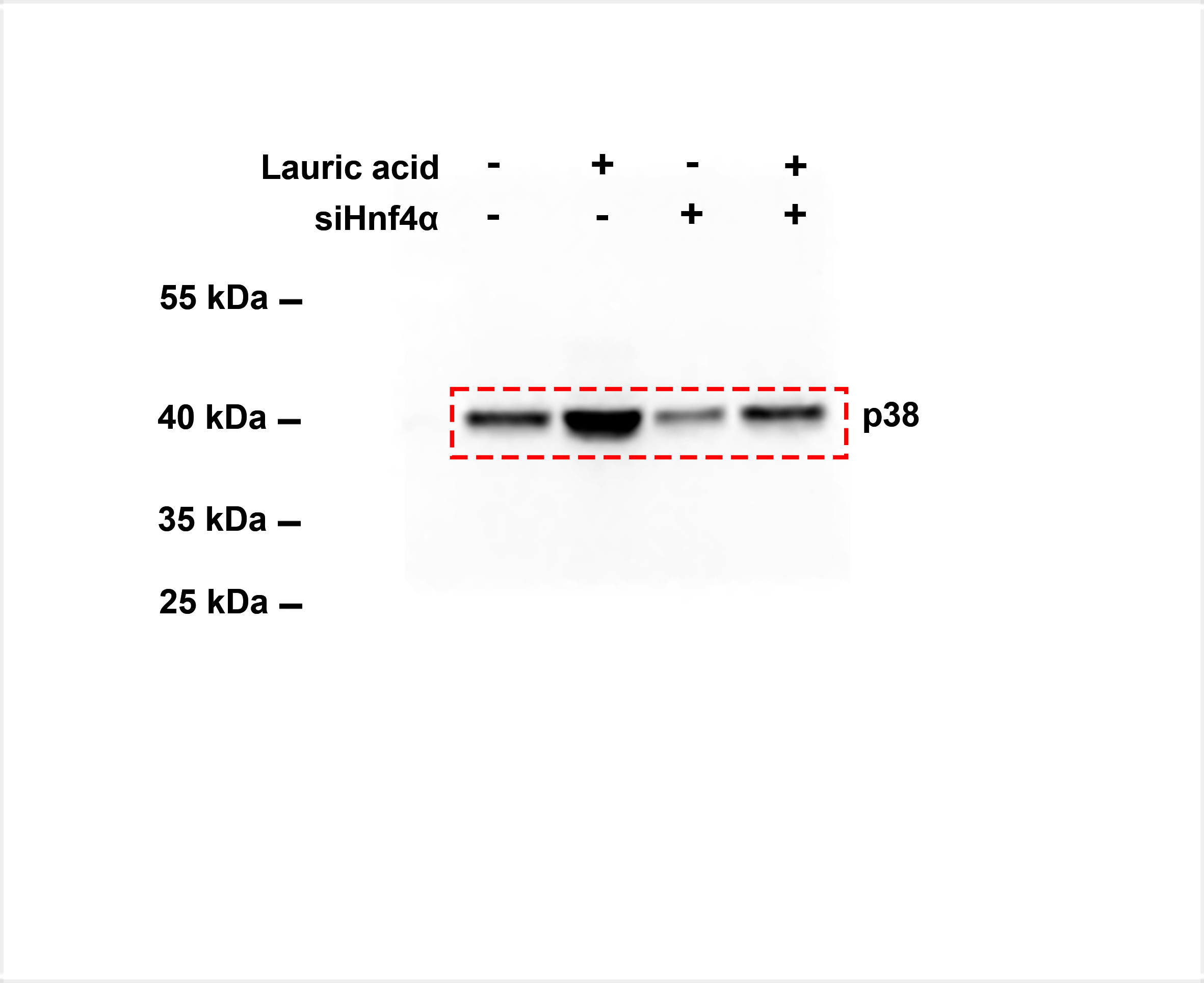

Supplement: Supplementary file 8 — Source Data Fig. 5 [file 44318_2023_20_MOESM8_ESM.zip › Figure 5/5L/p38.tif]

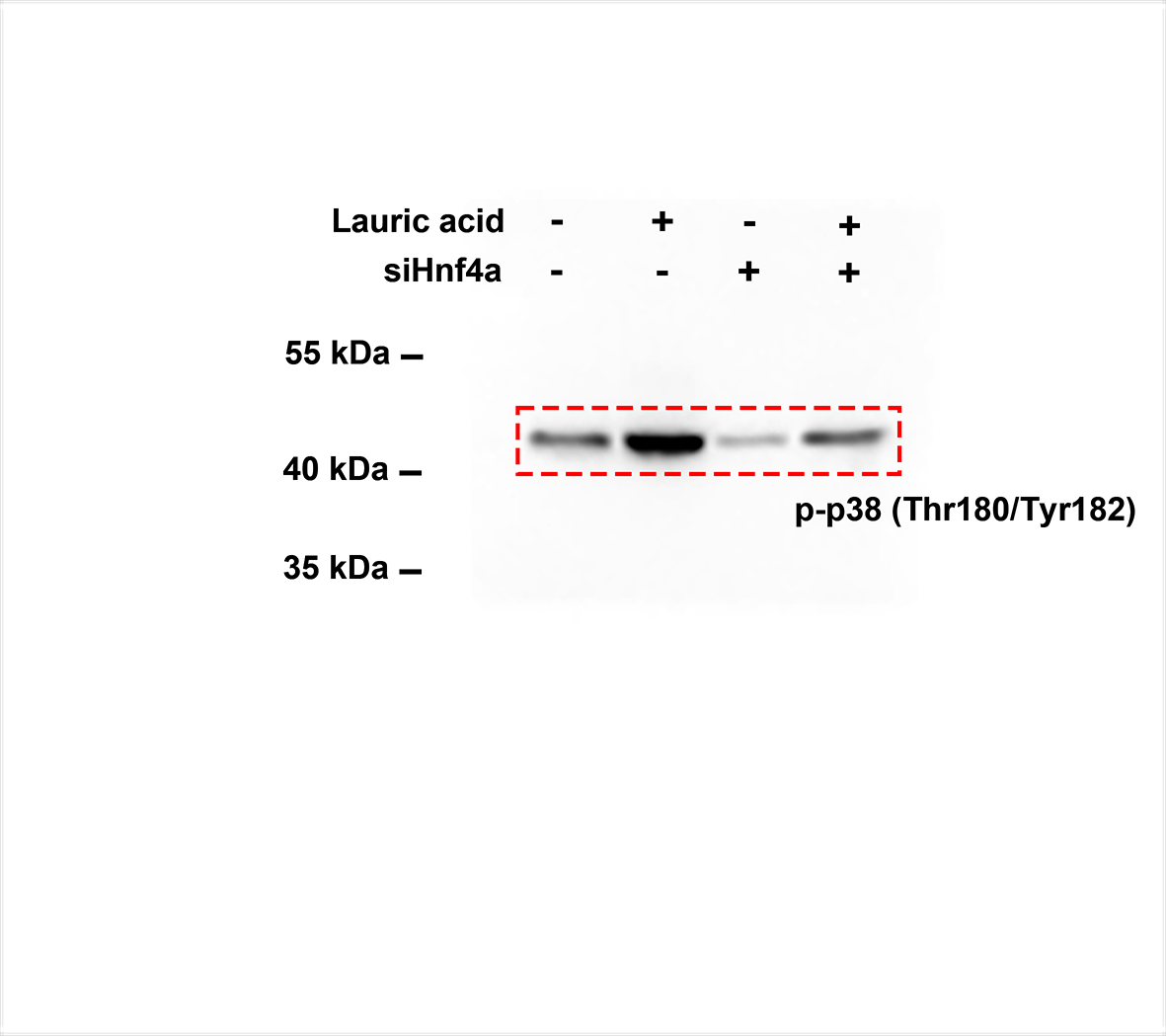

Supplement: Supplementary file 8 — Source Data Fig. 5 [file 44318_2023_20_MOESM8_ESM.zip › Figure 5/5L/p-p38(Thr180 Tyr182).tif]

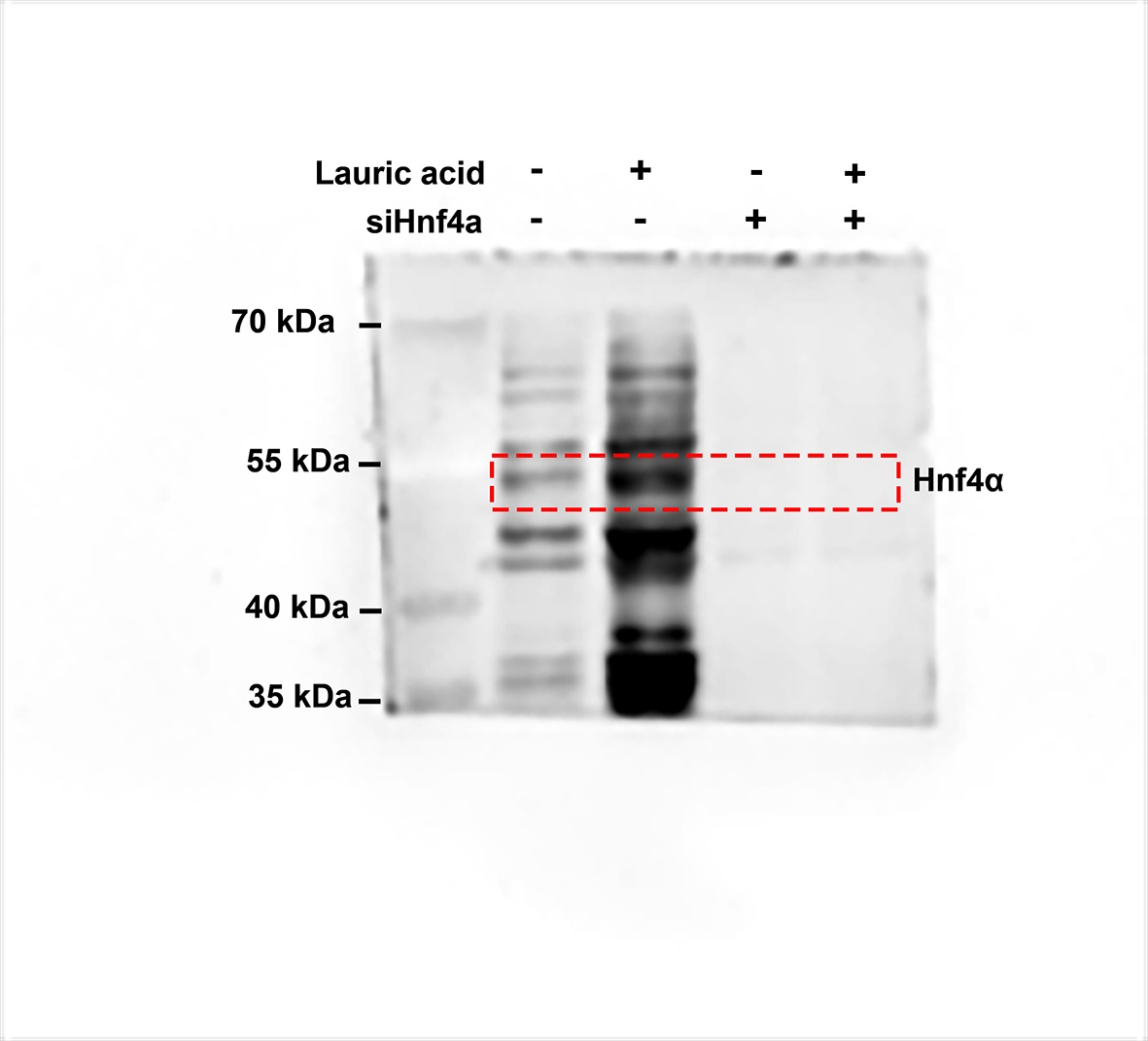

Supplement: Supplementary file 8 — Source Data Fig. 5 [file 44318_2023_20_MOESM8_ESM.zip › Figure 5/5L/Hnf4╬▒.tif]

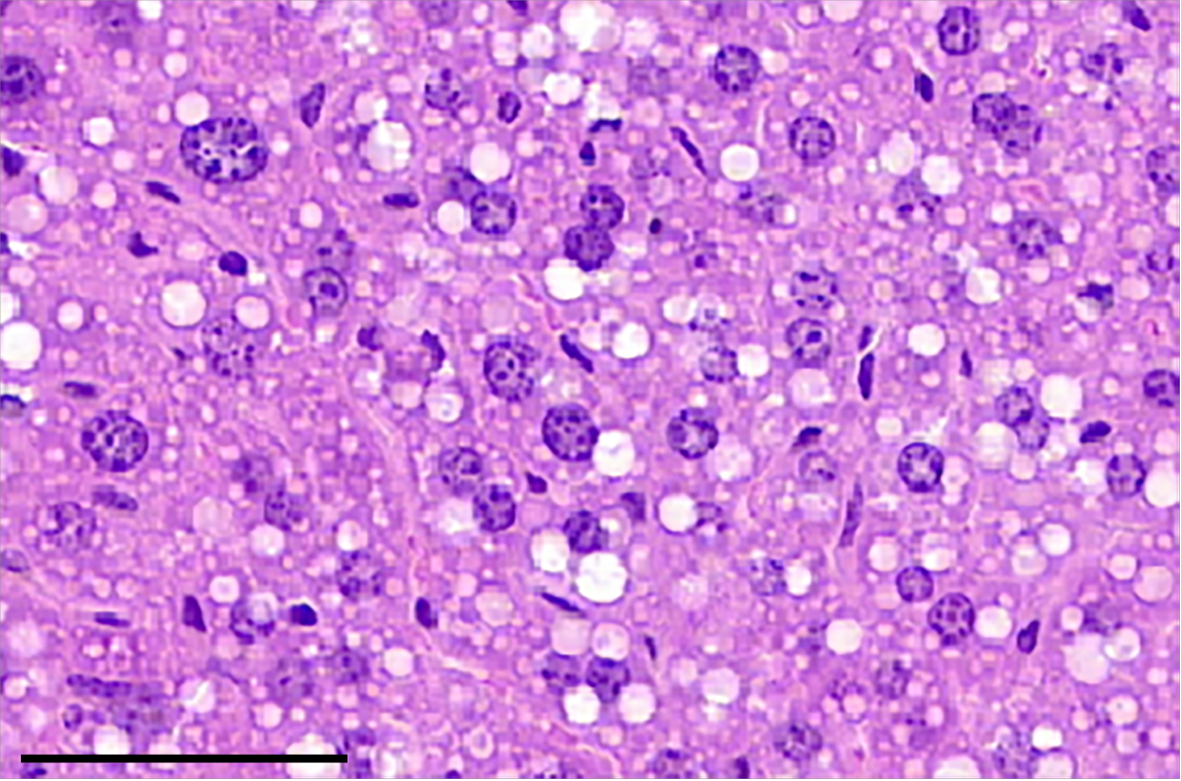

Supplement: Supplementary file 9 — Source Data Fig. 6 [file 44318_2023_20_MOESM9_ESM.zip › Figure 6/6F/HE_Acsm3 KO+Adezmapimod.tif]

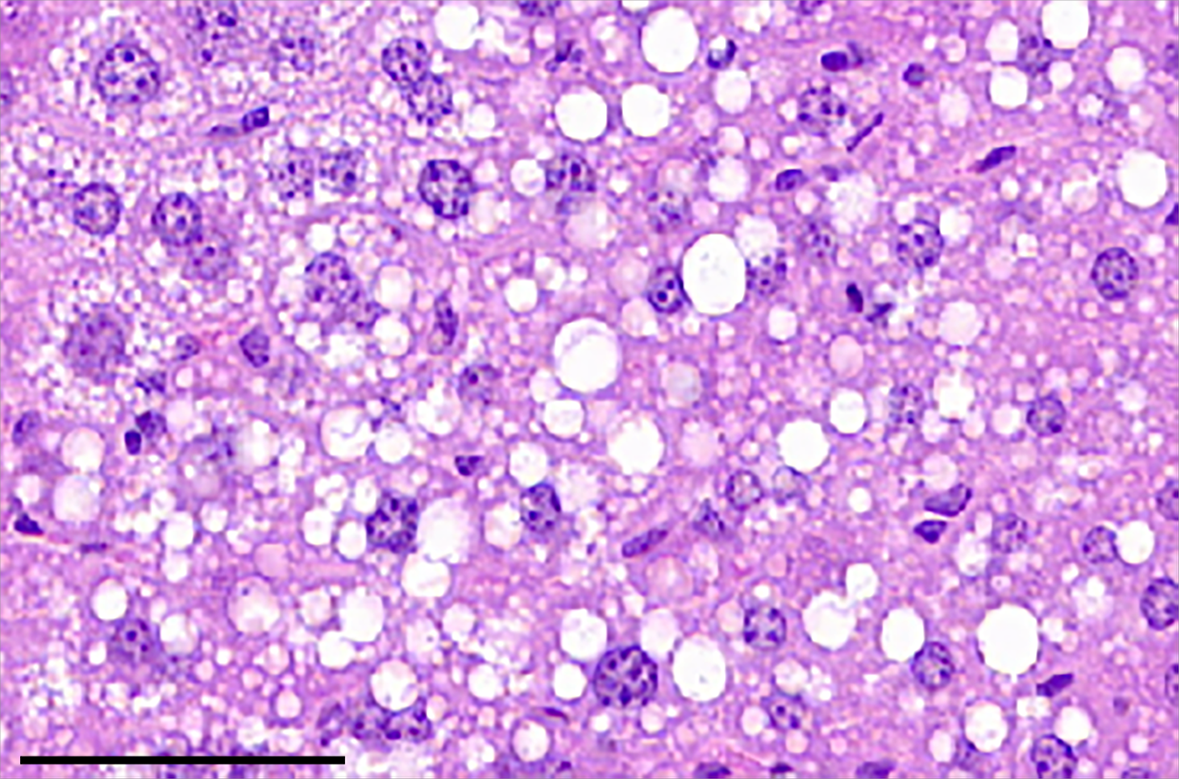

Supplement: Supplementary file 9 — Source Data Fig. 6 [file 44318_2023_20_MOESM9_ESM.zip › Figure 6/6F/HE_Acsm3 KO.tif]

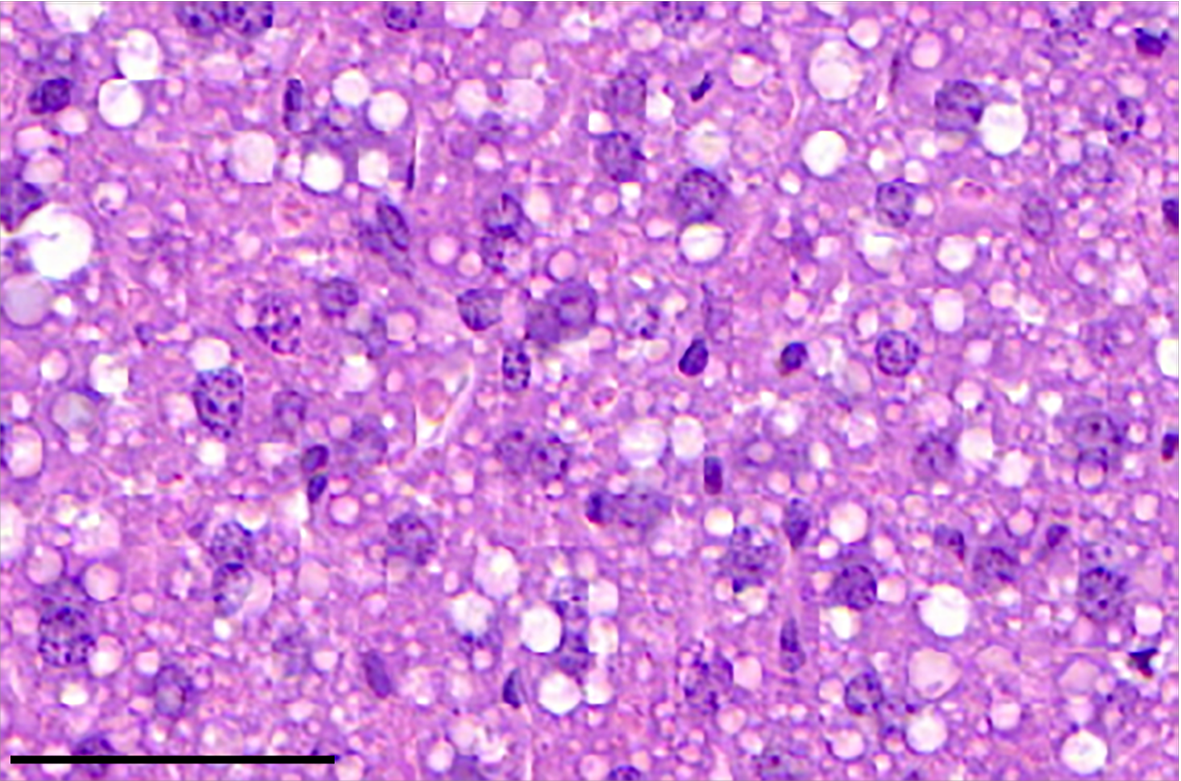

Supplement: Supplementary file 9 — Source Data Fig. 6 [file 44318_2023_20_MOESM9_ESM.zip › Figure 6/6F/HE_Control.tif]

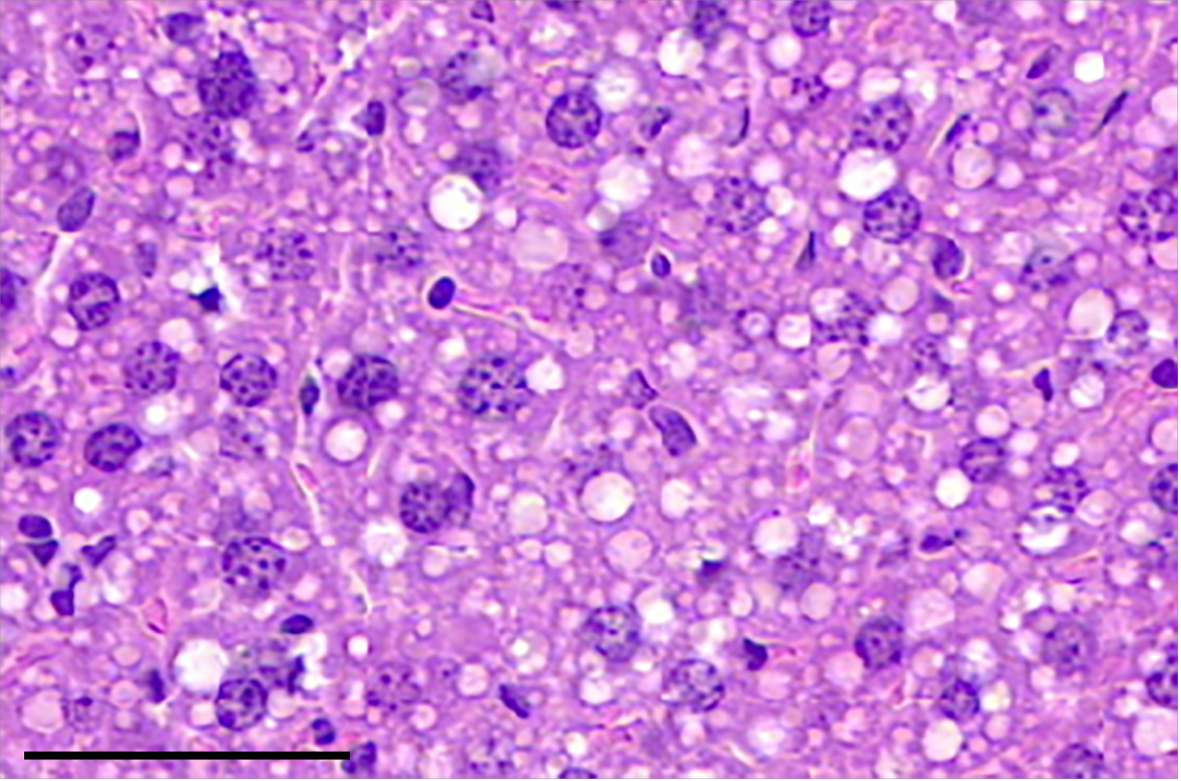

Supplement: Supplementary file 9 — Source Data Fig. 6 [file 44318_2023_20_MOESM9_ESM.zip › Figure 6/6F/HE_Control+Adezmapimod.tif]

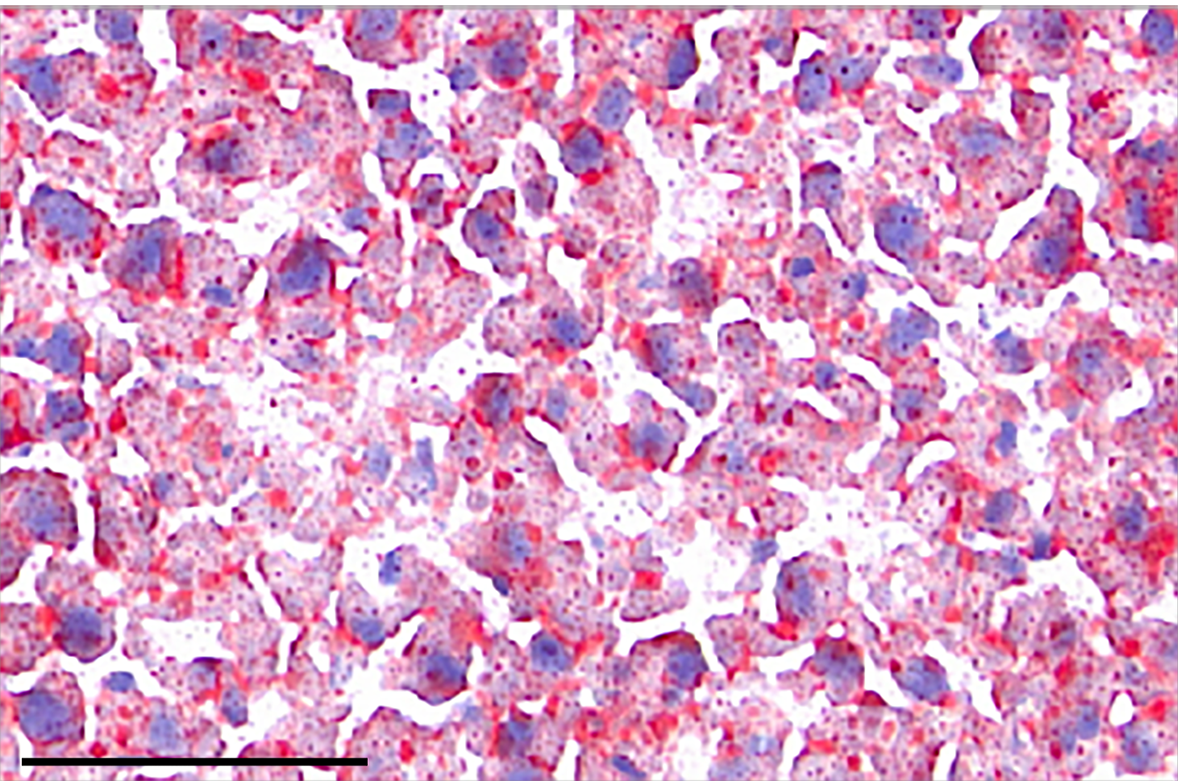

Supplement: Supplementary file 9 — Source Data Fig. 6 [file 44318_2023_20_MOESM9_ESM.zip › Figure 6/6G/Oil red O_Acsm3 KO+Adezmapimod.tif]

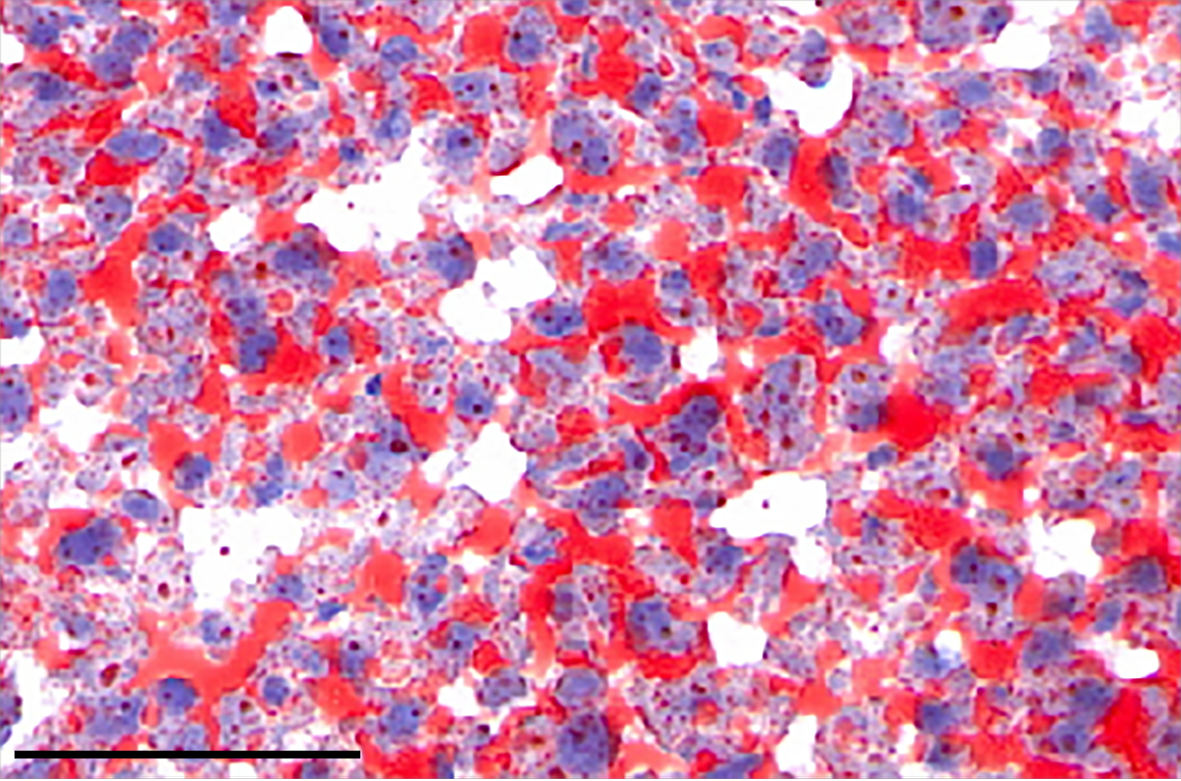

Supplement: Supplementary file 9 — Source Data Fig. 6 [file 44318_2023_20_MOESM9_ESM.zip › Figure 6/6G/Oil red O_Acsm3 KO.tif]

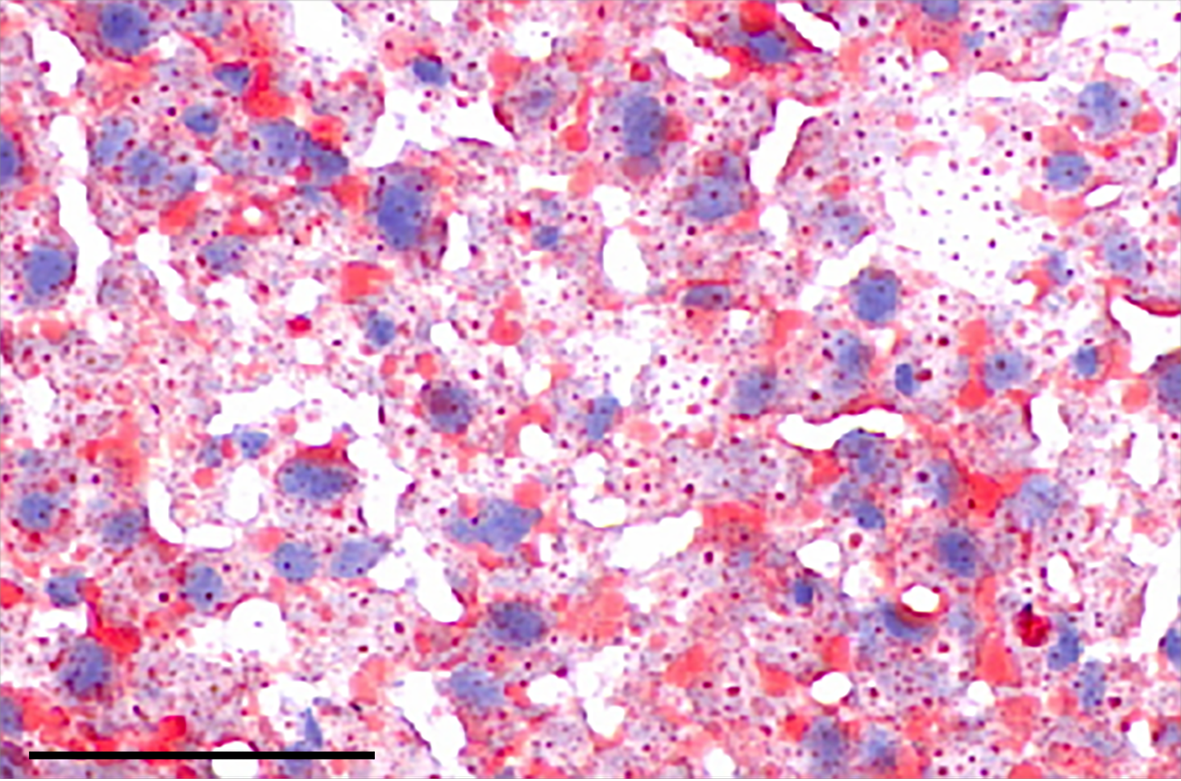

Supplement: Supplementary file 9 — Source Data Fig. 6 [file 44318_2023_20_MOESM9_ESM.zip › Figure 6/6G/Oil red O_Control+Adezmapimod.tif]

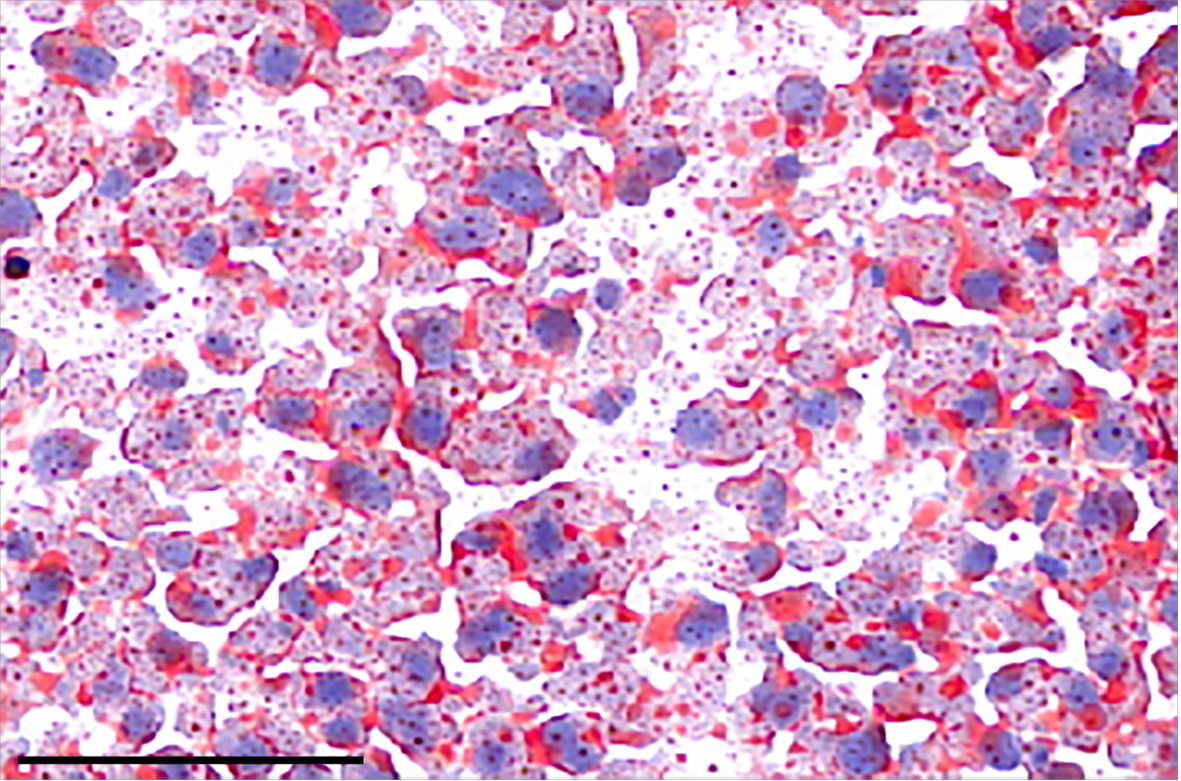

Supplement: Supplementary file 9 — Source Data Fig. 6 [file 44318_2023_20_MOESM9_ESM.zip › Figure 6/6G/Oil red O_Control.tif]

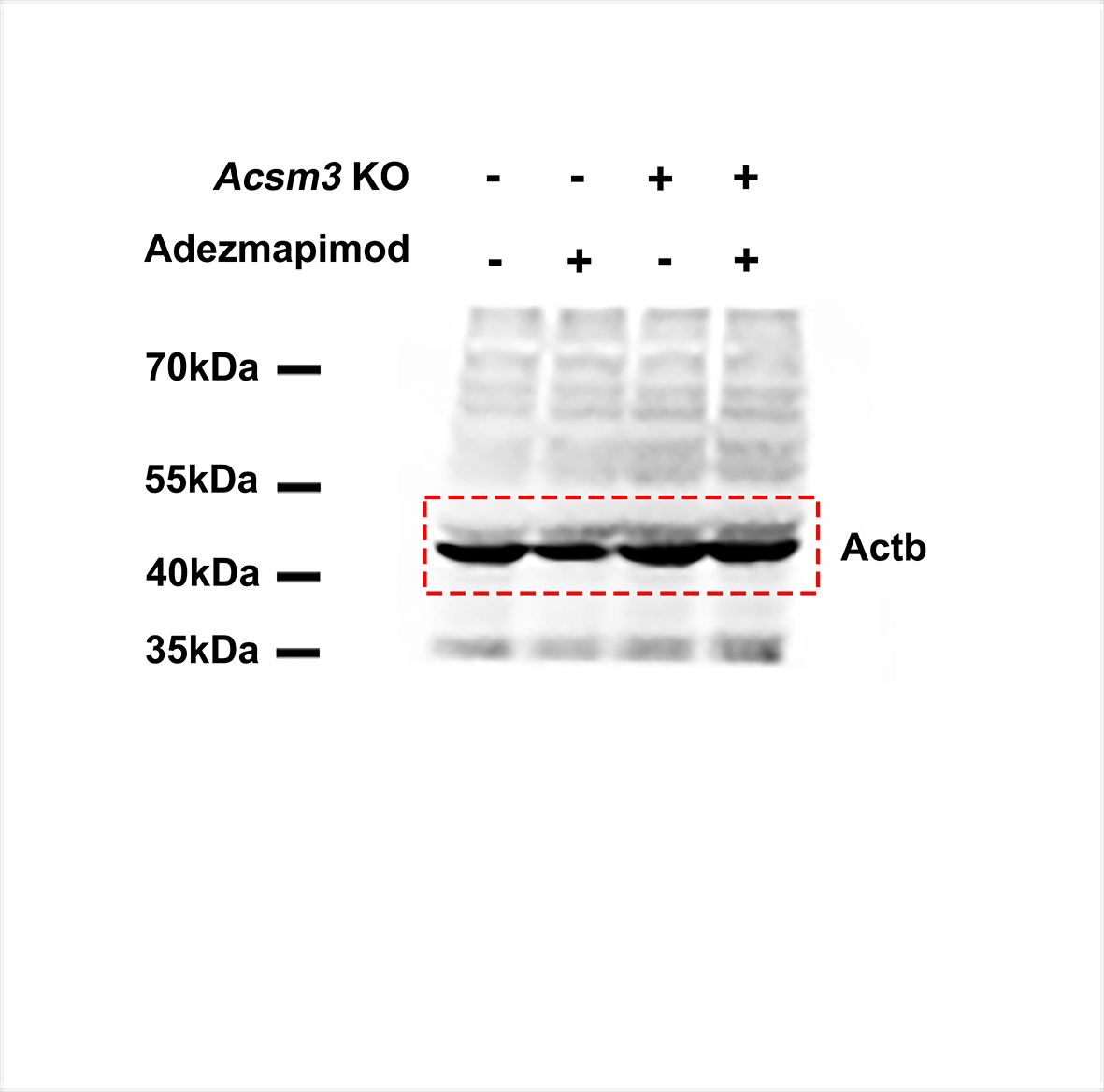

Supplement: Supplementary file 9 — Source Data Fig. 6 [file 44318_2023_20_MOESM9_ESM.zip › Figure 6/6B/Actb.tif]

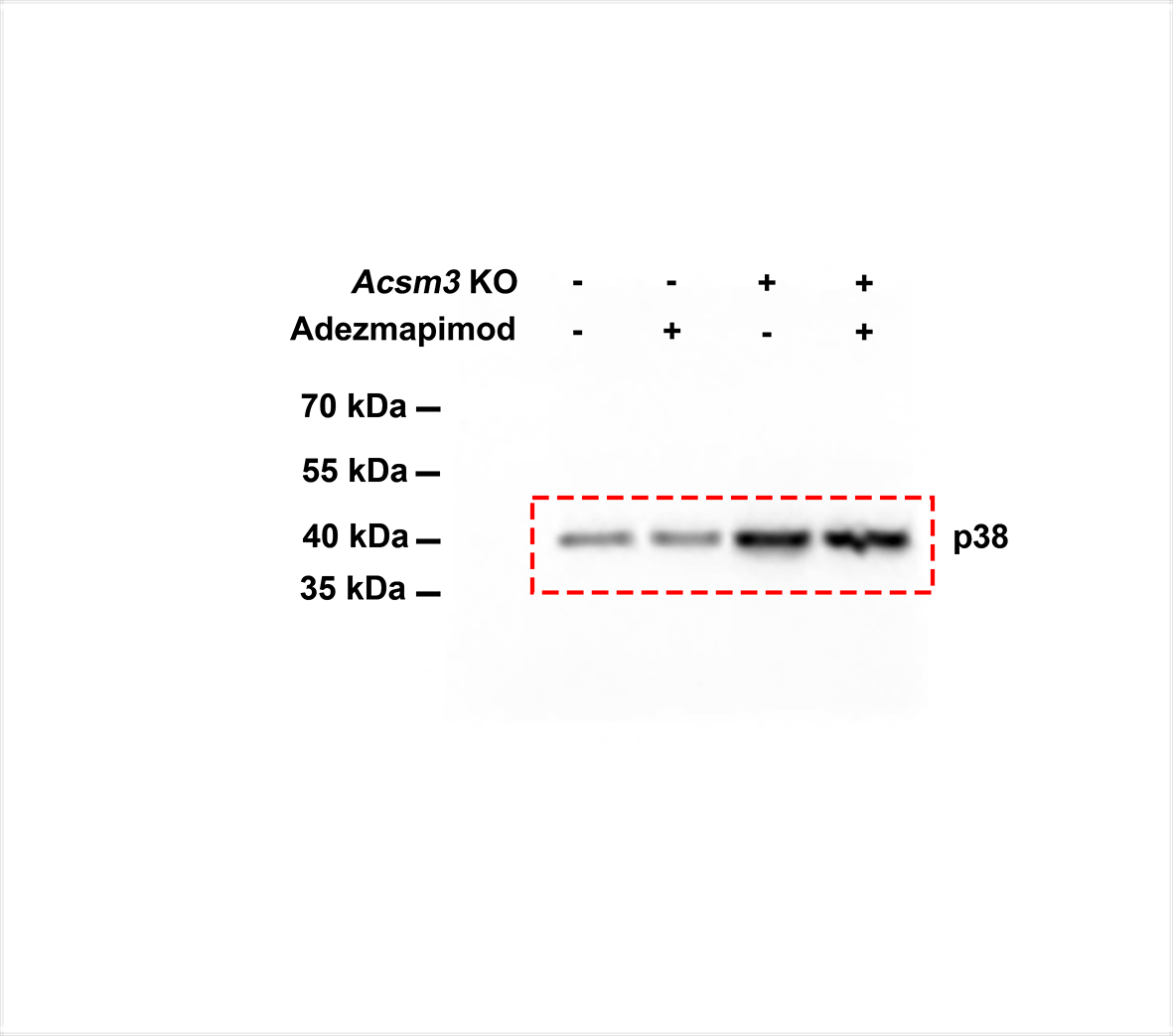

Supplement: Supplementary file 9 — Source Data Fig. 6 [file 44318_2023_20_MOESM9_ESM.zip › Figure 6/6B/p38.tif]

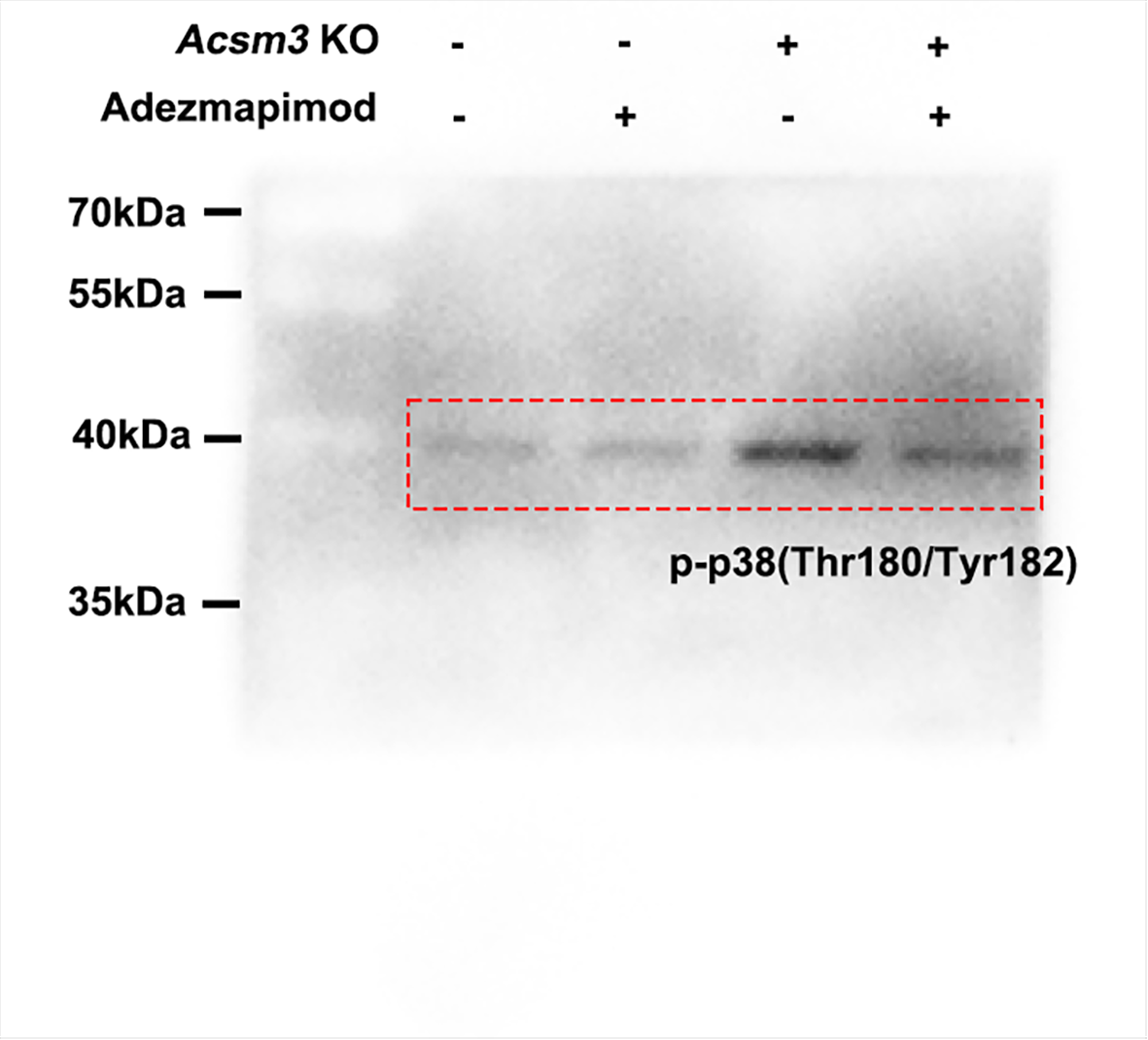

Supplement: Supplementary file 9 — Source Data Fig. 6 [file 44318_2023_20_MOESM9_ESM.zip › Figure 6/6B/p-p38(Thr180 Tyr182).tif]
