# Supplementary material for: Liver ACSM3 deficiency mediates metabolic syndrome via a lauric acid-HNF4α-p38 MAPK axis
Source: EMBO J. 2024 Jan 8;43(4):3. doi: 10.1038/s44318-023-00020-1 (PMC10897460; doi:10.1038/s44318-023-00020-1)
Supplement: Supplementary file 10 — Expanded View Figures [file 44318_2023_20_MOESM10_ESM.pdf]

Expanded View Figures

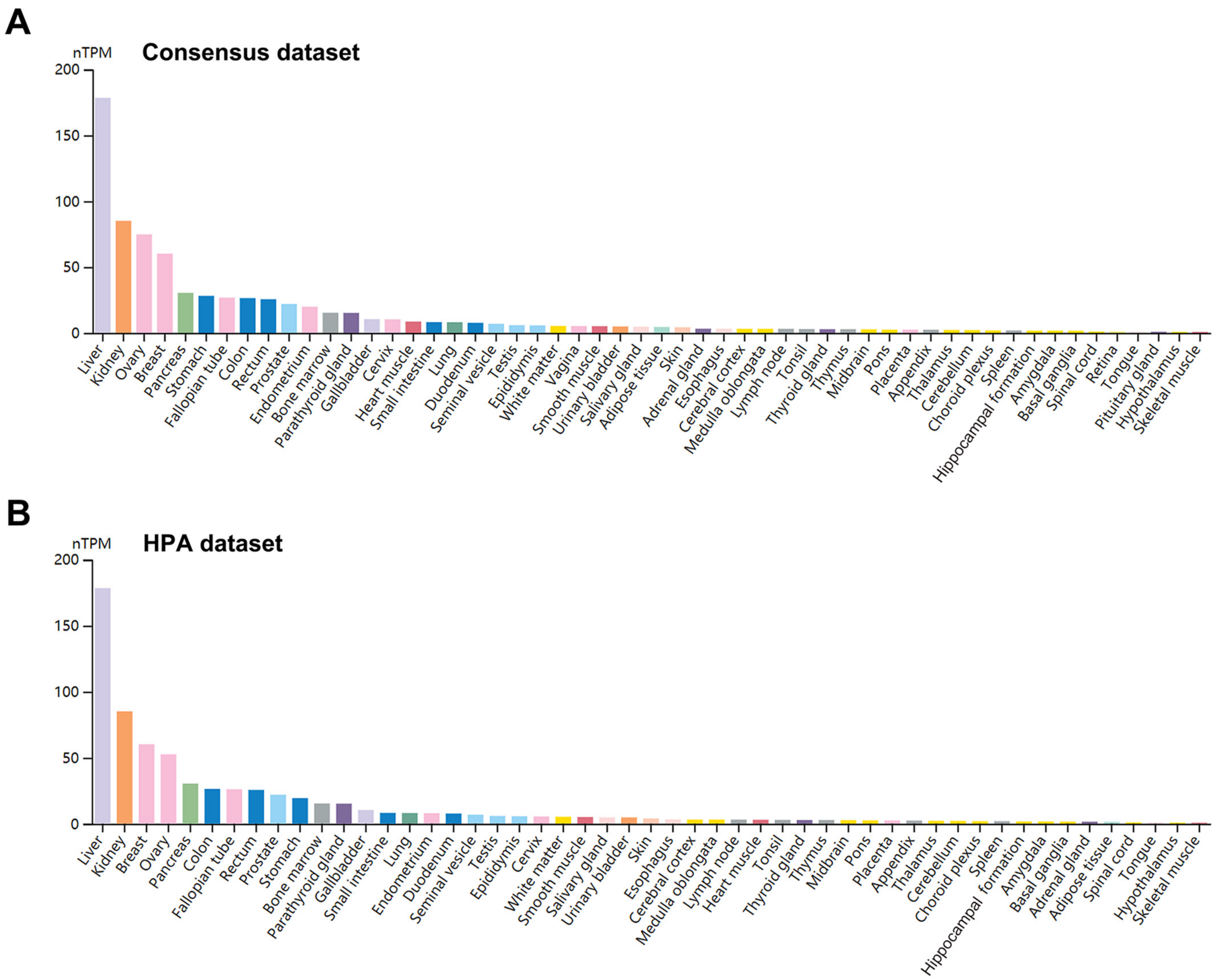

Figure EV1. Expression distributions of Acsm3.

(**A, B**) Consensus dataset and HPA dataset showing the expression of Acsm3 in different tissues in the human protein atlas database (https://www.proteinatlas.org/).

© The Author(s)

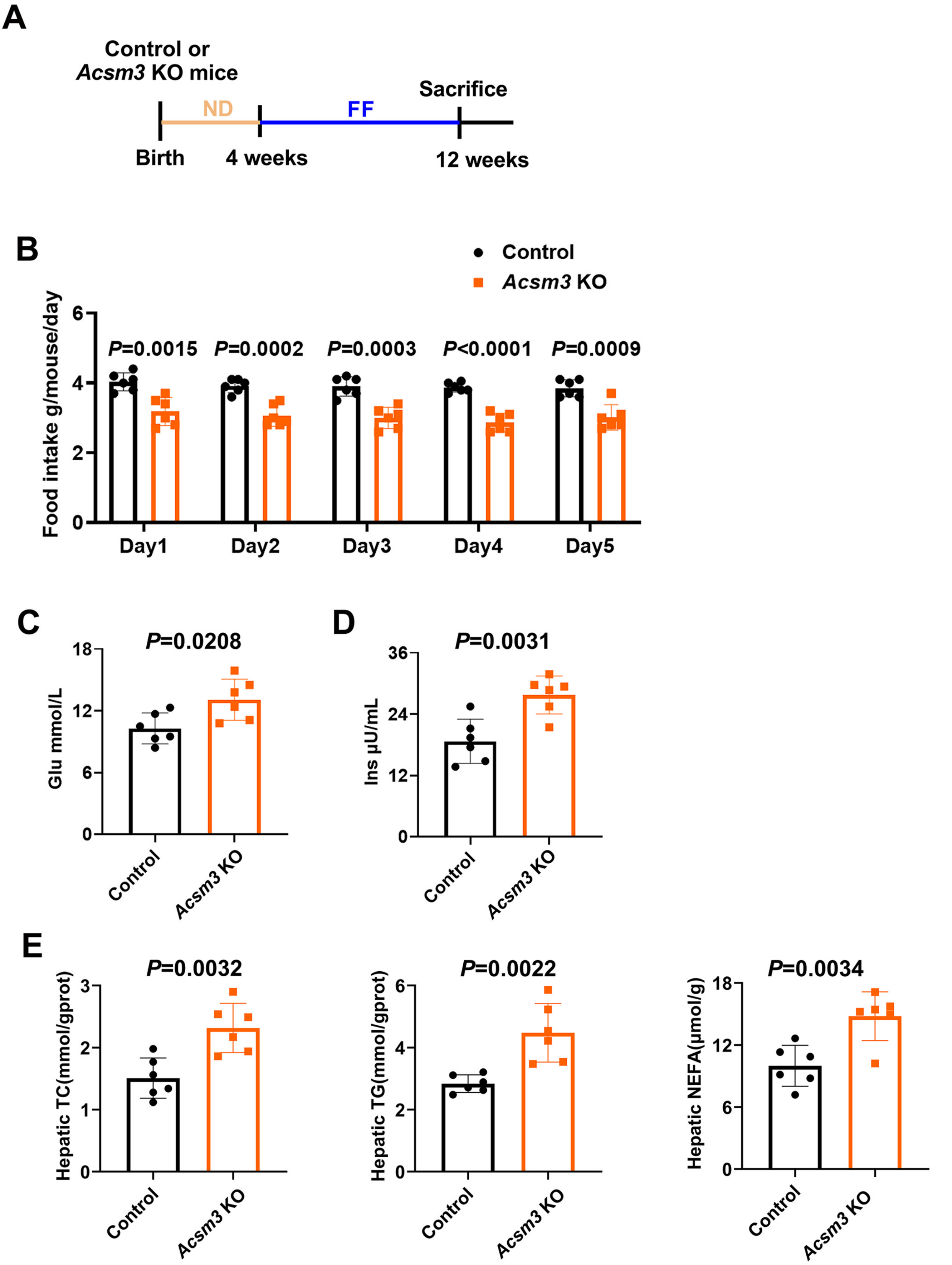
A
Control or
Acsm3 KO mice
ND
FF
Sacrifice
Birth
4 weeks
12 weeks
B
Control
Acsm3 KO
Food intake g/mouse/day
P=0.0015
P=0.0002
P=0.0003
P<0.0001
P=0.0009
Day1
Day2
Day3
Day4
Day5
C
P=0.0208
Glu mmol/L
Control
Acsm3 KO
D
P=0.0031
Ins μU/mL
Control
Acsm3 KO
E
P=0.0032
Hepatic TC(mmol/gprot)
P=0.0022
Hepatic TG(mmol/gprot)
P=0.0034
Hepatic NEFA(μmol/g)
Control
Acsm3 KO

 © The Author(s)

◀ **Figure EV2. *Acsm3* knockout mice under high-fat and high-fructose diet.**

(**A**) Timeline of *Acsm3* KO and control mice fed with an FF diet. (**B**) The average daily food intake of *Acsm3* KO and control mice for 5 consecutive days ($n = 6$ biologically independent samples in each group). Values were represented as the mean ± SD. Statistics were performed using Student's *t* test. (**C**, **D**) The fasting Glu (mmol/L), fasting Ins (μU/mL) of *Acsm3* KO and control mice. (**E**) The contents of hepatic TC (mmol/gprot), TG (mmol/gprot), and NEFA (μmol/g) in *Acsm3* KO and control mice ($n = 6$ biologically independent samples in each group). Values were represented as the mean ± SD. Statistics were performed using Student's *t* test.

© The Author(s)

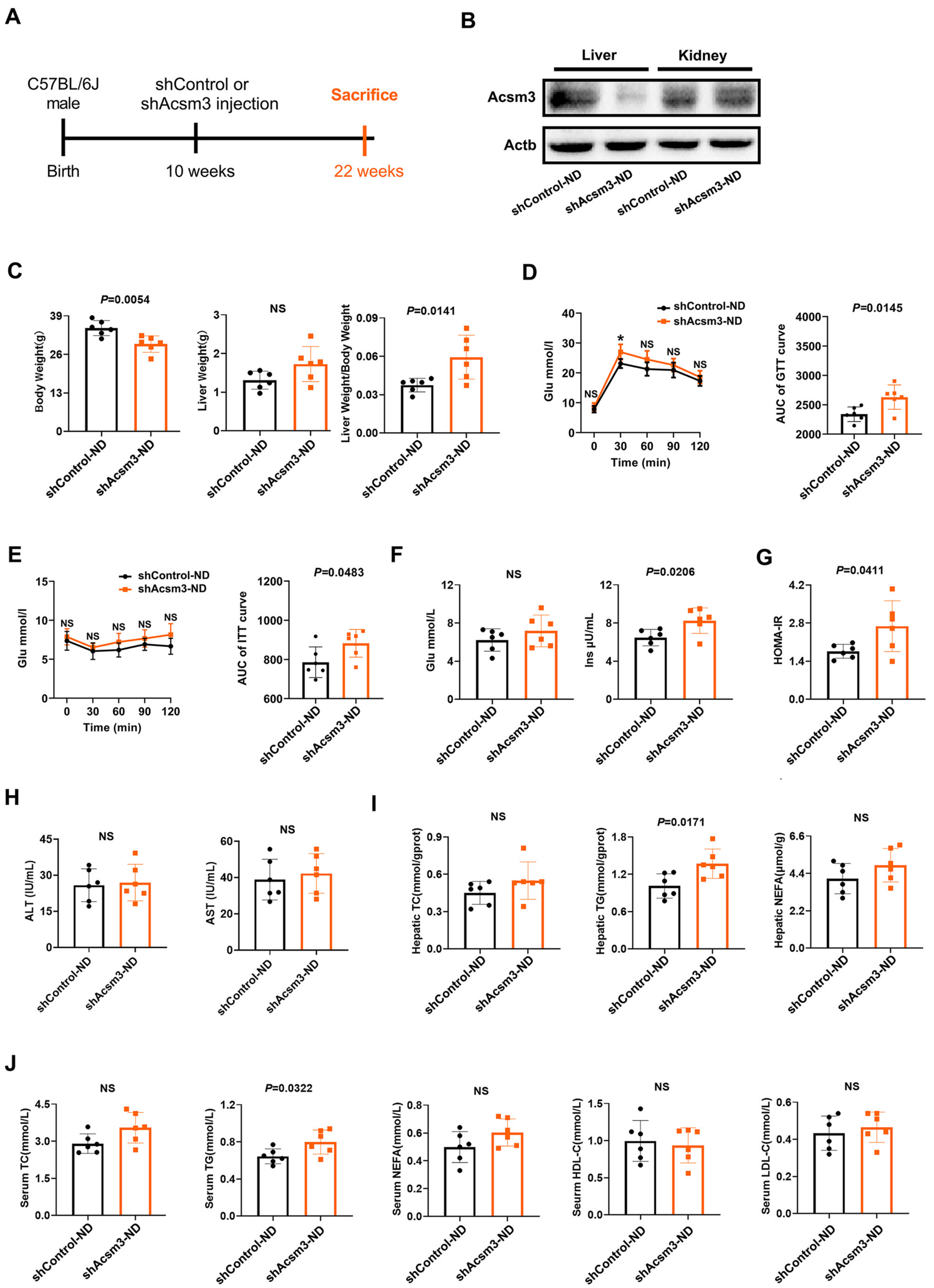
A
C57BL/6J male
shControl or shAcsm3 injection
Sacrifice
Birth
10 weeks
22 weeks
B
Liver
Kidney
Acsm3
Actb
shControl-ND
shAcsm3-ND
C
P=0.0054
Body Weight(g)
NS
Liver Weight(g)
P=0.0141
Liver Weight/Body Weight
D
shControl-ND
shAcsm3-ND
Glu mmol/l
Time (min)
P=0.0145
AUC of GTT curve
E
AUC of ITT curve
P=0.0483
F
Glu mmol/L
Ins μU/mL
P=0.0206
G
HOMA-IR
P=0.0411
H
ALT (IU/mL)
AST (IU/mL)
I
Hepatic TC(mmol/gprot)
Hepatic TG(mmol/gprot)
P=0.0171
Hepatic NEFA(μmol/g)
J
Serum TC(mmol/L)
Serum TG(mmol/L)
P=0.0322
Serum NEFA(mmol/L)
Seurm HDL-C(mmol/L)
Serum LDL-C(mmol/L)

 © The Author(s)

Figure EV3. Acsm3 liver-specific knocked down mice under normal diet.

(**A**) Timeline of shAcsm3 mice (Adeno-associated virus 8 (AAV8) injection induced liver-specific knockdown of Acsm3 in mice) and shControl mice under ND. (**B**) Western blot showing the relative expression of Acsm3 in the liver and kidney of shAcsm3 and shControl mice. (**C**) The body weights (g), liver weights (g), and liver weight/body weight of shAcsm3 and shControl mice ($n = 6$ biologically independent samples in each group). Values were represented as the mean ± SD. Statistics were performed using Student's *t* test. (**D**, **E**) GTT, ITT, and respective AUC of shAcsm3 and shControl mice ($n = 6$ biologically independent samples in each group). Values were represented as the mean ± SD. Statistics were performed using Student's *t* test. *P* values were denoted by asterisks: $^*P < 0.05$. (**F**, **G**) The fasting Glu (mmol/L), fasting Ins (μU/mL), and HOMA-IR indexes of shAcsm3 and shControl mice ($n = 6$ biologically independent samples in each group). HOMA-IR = fasting Glu (mmol/L) × fasting Ins (μU/mL)/22.5. Values were represented as the mean ± SD. Statistics were performed using Student's *t* test. (**H**) The contents (IU/mL) of serum ALT and AST in shAcsm3 and shControl mice ($n = 6$ biologically independent samples in each group). Values were represented as the mean ± SD. Statistics were performed using Student's *t* test. (**I**) The contents of hepatic TC (mmol/gprot), TG ((mmol/gprot)), and NEFA (μmol/g) in shAcsm3 and shControl mice ($n = 6$ biologically independent samples in each group). Values were represented as the mean ± SD. Statistics were performed using Student's *t* test. (**J**) The contents (mmol/L) of serum TC, TG, NEFA, HDL-C, and LDL-C in shAcsm3 and shControl mice ($n = 6$ biologically independent samples in each group). Values were represented as the mean ± SD. Statistics were performed using Student's *t* test.

© The Author(s)

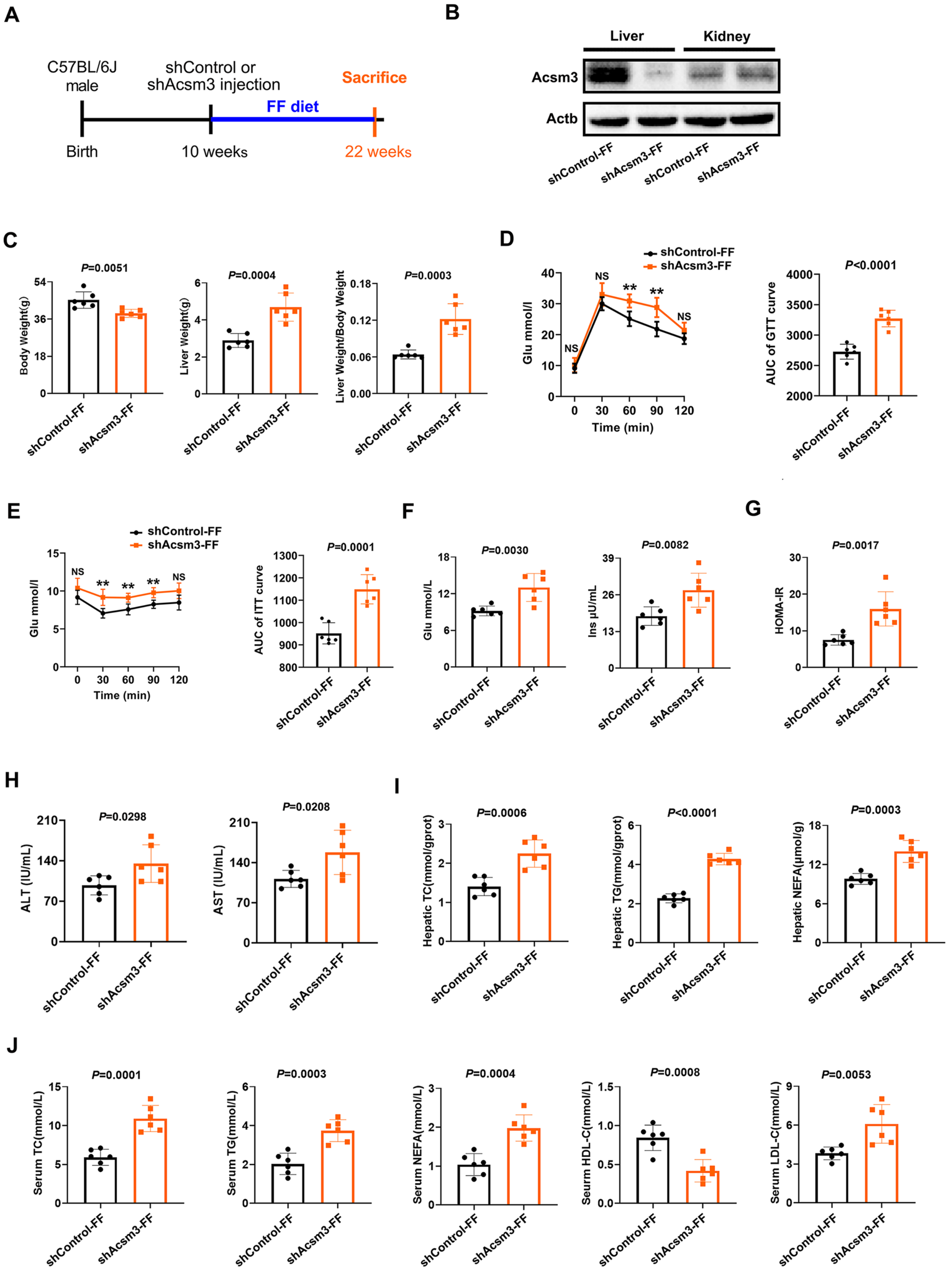
A
C57BL/6J
male
shControl or
shAcsm3 injection
Sacrifice
FF diet
Birth
10 weeks
22 weeks
B
Liver
Kidney
Acsm3
Actb
shControl-FF
shAcsm3-FF
shControl-FF
shAcsm3-FF
C
P=0.0051
Body Weight(g)
P=0.0004
Liver Weight(g)
P=0.0003
Liver Weight/Body Weight
D
shControl-FF
shAcsm3-FF
Glu mmol/l
Time (min)
P<0.0001
AUC of GTT curve
E
shControl-FF
shAcsm3-FF
Glu mmol/l
Time (min)
P=0.0001
AUC of ITT curve
F
P=0.0030
Glu mmol/L
P=0.0082
Ins μU/mL
G
P=0.0017
HOMA-IR
H
P=0.0298
ALT (IU/mL)
P=0.0208
AST (IU/mL)
I
P=0.0006
Hepatic TC(mmol/gprot)
P<0.0001
Hepatic TG(mmol/gprot)
P=0.0003
Hepatic NEFA(μmol/g)
J
P=0.0001
Serum TC(mmol/L)
P=0.0003
Serum TG(mmol/L)
P=0.0004
Serum NEFA(mmol/L)
P=0.0008
Serum HDL-C(mmol/L)
P=0.0053
Serum LDL-C(mmol/L)

 © The Author(s)

Figure EV4. Acsm3 liver-specific knocked down mice under FF diet.

(**A**) Timeline of shAcsm3 mice and shControl mice fed with an FF diet. (**B**) Western blot showing the relative expression of Acsm3 in the liver and kidney of shAcsm3 and shControl mice. (**C**) The body weights (g), liver weights (g), and liver weight/body weight of shAcsm3 and shControl mice ($n = 6$ biologically independent samples in each group). Values were represented as the mean ± SD. Statistics were performed using Student's *t* test. (**D**, **E**) GTT, ITT, and respective AUC of shAcsm3 and shControl mice ($n = 6$ biologically independent samples in each group). Values were represented as the mean ± SD. Statistics were performed using Student's *t* test. *P* values were denoted by asterisks: **$P < 0.01$. (**F**, **G**) The fasting Glu (mmol/L), fasting Ins (μU/mL), and HOMA-IR indexes of shAcsm3 and shControl mice ($n = 6$ biologically independent samples in each group). HOMA-IR = fasting Glu (mmol/L) × fasting Ins (μU/mL)/22.5. Values were represented as the mean ± SD. Statistics were performed using Student's *t* test. (**H**) The contents (IU/mL) of serum ALT and AST in shAcsm3 and shControl mice ($n = 6$ biologically independent samples in each group). Values were represented as the mean ± SD. Statistics were performed using Student's *t* test. (**I**) The contents of hepatic TC (mmol/gprot), TG ((mmol/gprot)), and NEFA (μmol/g) in shAcsm3 and shControl mice ($n = 6$ biologically independent samples in each group). Values were represented as the mean ± SD. Statistics were performed using Student's *t* test. (**J**) The contents (mmol/L) of serum TC, TG, NEFA, HDL-C, and LDL-C in shAcsm3 and shControl mice ($n = 6$ biologically independent samples in each group). Values were represented as the mean ± SD. Statistics were performed using Student's *t* test.

© The Author(s)

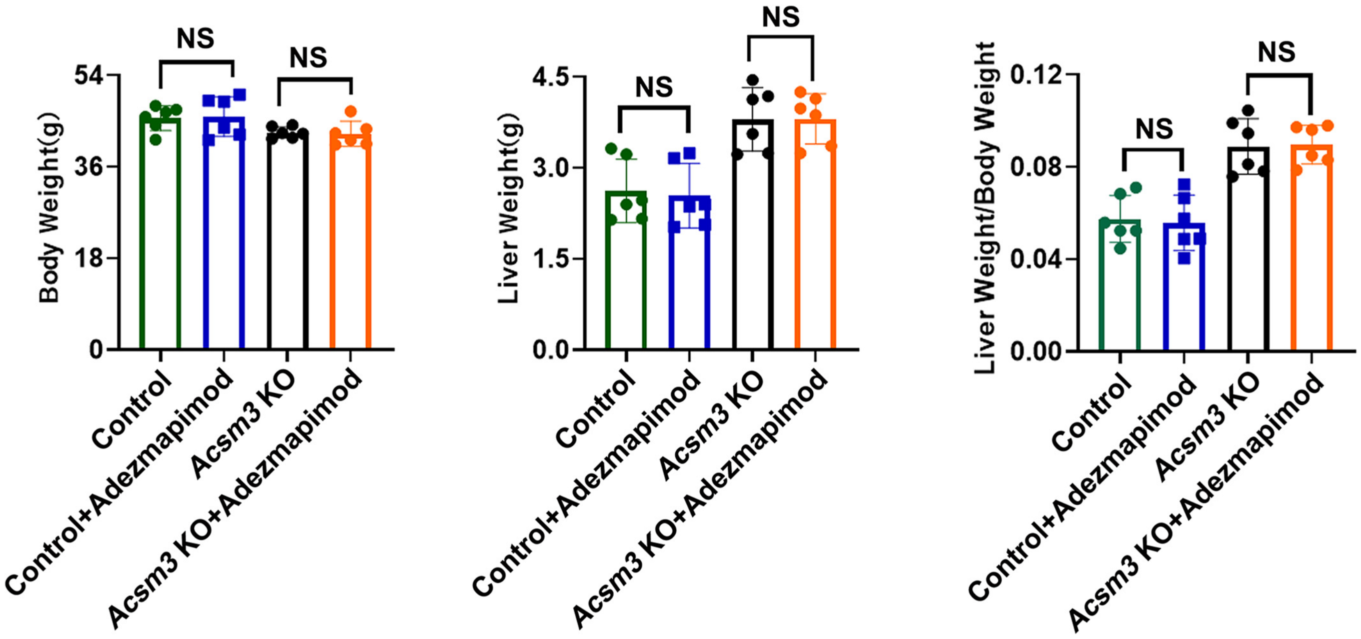

Figure EV5. The body weights (g), liver weights (g), and liver weight/body weight of *Acsm3* KO and control mice treated with adezmapimod or not.

Values were represented as the mean ± SD ($n = 6$ biologically independent samples in each group). Statistics were performed using Student's t test.

 © The Author(s)
